# Delivery of oncolytic vaccinia virus by matched allogeneic stem cells overcomes critical innate and adaptive immune barriers

**DOI:** 10.1186/s12967-019-1829-z

**Published:** 2019-03-27

**Authors:** Dobrin D. Draganov, Antonio F. Santidrian, Ivelina Minev, Duong Nguyen, Mehmet Okyay Kilinc, Ivan Petrov, Anna Vyalkova, Elliot Lander, Mark Berman, Boris Minev, Aladar A. Szalay

**Affiliations:** 1 Calidi Biotherapeutics, San Diego, CA 92121 USA; 2 Institute of Biochemistry, Biocentre, University of Wuerzburg, Am Hubland, 97070 Würzburg, Germany; 3 Radiation Oncology, Moores Cancer Center, University of California San Diego, La Jolla, San Diego, CA 92037 USA; 4 California Stem Cell Treatment Center, Rancho Mirage, CA 92270 USA

**Keywords:** Vaccinia, Cancer, Stem Cells, Oncolysis, Oncolytic virus, Virotherapy, Immunity, Immunotherapy

## Abstract

**Background:**

Previous studies have identified IFNγ as an important early barrier to oncolytic viruses including vaccinia. The existing innate and adaptive immune barriers restricting oncolytic virotherapy, however, can be overcome using autologous or allogeneic mesenchymal stem cells as carrier cells with unique immunosuppressive properties.

**Methods:**

To test the ability of mesenchymal stem cells to overcome innate and adaptive immune barriers and to successfully deliver oncolytic vaccinia virus to tumor cells, we performed flow cytometry and virus plaque assay analysis of ex vivo co-cultures of stem cells infected with vaccinia virus in the presence of peripheral blood mononuclear cells from healthy donors. Comparative analysis was performed to establish statistically significant correlations and to evaluate the effect of stem cells on the activity of key immune cell populations.

**Results:**

Here, we demonstrate that adipose-derived stem cells (ADSCs) have the potential to eradicate resistant tumor cells through a combination of potent virus amplification and sensitization of the tumor cells to virus infection. Moreover, the ADSCs demonstrate ability to function as a virus-amplifying Trojan horse in the presence of both autologous and allogeneic human PBMCs, which can be linked to the intrinsic immunosuppressive properties of stem cells and their unique potential to overcome innate and adaptive immune barriers. The clinical application of ready-to-use ex vivo expanded allogeneic stem cell lines, however, appears significantly restricted by patient-specific allogeneic differences associated with the induction of potent anti-stem cell cytotoxic and IFNγ responses. These allogeneic responses originate from both innate (NK)- and adaptive (T)- immune cells and might compromise therapeutic efficacy through direct elimination of the stem cells or the induction of an anti-viral state, which can block the potential of the Trojan horse to amplify and deliver vaccinia virus to the tumor.

**Conclusions:**

Overall, our findings and data indicate the feasibility to establish simple and informative assays that capture critically important patient-specific differences in the immune responses to the virus and stem cells, which allows for proper patient-stem cell matching and enables the effective use of off-the-shelf allogeneic cell-based delivery platforms, thus providing a more practical and commercially viable alternative to the autologous stem cell approach.

**Electronic supplementary material:**

The online version of this article (10.1186/s12967-019-1829-z) contains supplementary material, which is available to authorized users.

## Background

Successful virotherapy of cancer is critically dependent on the ability of oncolytic viruses like vaccinia to overcome multiple defense barriers including the complement/antibody-mediated neutralization [1], the interferon-induced anti-viral state [2–5], as well as the innate and adaptive anti-viral immune mechanisms mediated by NK and T cells, respectively. Mesenchymal stem cells (MSC) represent a promising delivery vehicle that protects vaccinia virus from the effects of complement/neutralizing antibodies [6], while also having the unique ability to home to sites of inflammation and tumor growth [7]. This therapeutic platform also takes advantage of the immunosuppressive properties of MSC [8–10], in particular, their ability to survive undetected in allogeneic settings and to transiently counteract the innate and adaptive anti-viral immunity [11], thus enabling rapid virus spread and colonization of the tumor [12, 13].

We therefore investigated the potential and feasibility of using ex vivo expanded mesenchymal stem cells as a delivery vehicle for oncolytic vaccinia virus. Mesenchymal stem cells can be expanded from various sources including adult bone marrow [14], adipose tissue [15], blood, and dental pulp or neonatal umbilical cord, placenta etc. [16–19], and are known to possess potent immunosuppressive properties mediated by IDO [20–22], PGE [23–25], Adenosine [26], TGFβ [10, 27, 28], VEGF [29, 30], HGF [31–34], iNOS [35–38], IL-10 [27], HLA-G5 [39], and Galectins [40], regardless of the source of origin [16, 29, 41]. Adipose-derived stem cells (ADSCs) isolated from the stromal vascular fraction (SVF) of liposuction aspirates are particularly useful because they are easily available and can be efficiently expanded in culture for different applications [42–45]. Our studies indicate that these carrier cells possess impressive virus amplification potential and the capacity to immunosuppress both the innate and adaptive arms of anti-viral immunity.

The immunosuppressive properties of mesenchymal stem cells have been investigated extensively and supported by clinical data demonstrating their ability to avoid immune recognition and survive even in MHC-mismatched allogeneic recipients [11, 46–48]. What remains still unclear, however, is how the immunosuppressive properties of MSCs will be affected by the progression of vaccinia virus infection especially in allogeneic MHC-mismatched settings. Importantly, MSC-mediated immunosuppression is subject to differential modulation by both type I [36, 49] and II interferons [22, 50–55], which are similarly involved in the control of vaccinia virus infection [2, 3, 56, 57]. While type I interferons are secreted by any cell type in response to viral infection, including the MSC, Interferon gamma (IFNγ) is a type II interferon that is produced by a limited number of immune cells like NK, NKT and T cells in response to virus infection [58] or, as we demonstrate, the carrier stem cells, particularly in some unfavorable allogeneic MHC-mismatch settings. These conflicting roles of IFNγ to limit virus spread through the induction of an anti-viral state but at the same time to stimulate MSC-mediated immunosuppression counteracting anti-viral immunity might prove to be a critically important determinant of therapeutic success.

We hypothesized that the complex interplay between vaccinia virus, ADSCs and immune cells could have significant impact on the ability of stem cells to function as a Trojan horse that can amplify and deliver oncolytic vaccinia virus. Here we show that interferons protect stem cells against vaccinia virus infection but compromise their function as a Trojan horse. The IFNγ responses to the virus and allogeneic stem cells alone or in combination, however, appear to be highly variable and patient-specific. Our studies indicate that these differences can be associated with subtle allogeneic NK- and T cell-mediated cytotoxic and cytokine responses that can result in the inactivation or complete rejection of the Trojan horse. These findings have significant implications for the development of cell-based delivery platforms for oncolytic viruses and suggest the need for proper screening and patient-specific matching to enable the successful use of off-the-shelf allogeneic cell carriers, as opposed to the more expensive personalized autologous stem cell approach.

## Materials and methods

### Cell lines, cytokines, and viruses

B16 F10 melanoma, A549 lung carcinoma, and CV-1 monkey kidney cells were obtained from Dr. Boris Minev, and K562 cells were a kind gift from Albert Perez-Ladaga, PhD. Cells were propagated in DMEM (B16, A549) from Gibco (Cat#: 11960069) or RPMI 1640 (K562) from Gibco (Cat: 21870092) supplemented with 10% Fetal Bovine Serum (Omega Scientific, FB-02, USDA certified, heat inactivated), 2 mM l-Glutamine (ThermoFisher Scientific, 25030081, 100×) and Penicillin/Streptomycin (Life Technologies, 15140122, 100×). Human IFNγ (Peprotech, cat# AF3000220UG, 20 mg lyophilized, diluted to 20 μg/ml approx. 1000× stock in 1×PBS supplemented with 0.1% FBS, stored at − 80 °C) and IFNβ (Peprotech, cat# AF30002B5UG, 5 μg lyophilized, diluted to 5 μg/ml approx. 1000× stock in 1×PBS supplemented with 0.1% FBS, stored at − 80 °C) were added to ADSCs for 24 h 1 to 3 days prior to virus infection. Sucrose gradient purified WT1/ACAM2000 and L14 (TK-inserted Turbo-FP635 engineered LIVP strain) vaccinia viruses were obtained from StemVac GmbH, Bernried, Germany. In some experiments the virus and ADSCs were pre-infected for 1 h with constant agitation on an orbital shaker at 37 °C (incubator) before adding them to PBMCs or tumor cell co-cultures.

### Adipose-derived stem cells isolation and culture

Non-cancer donor SVF and PBMC were obtained as part of an IRB-approved protocol after informed written consent (International Cell Surgical Society; IRB# ICSS-2016-024). Fresh SVF fractions were plated to attach overnight and next day were washed to remove unattached cells and debris. Media was changed every 3–4 days until the mesenchymal stem cells start to grow and reach 80% confluency. Cell were expanded to 80% confluency and passaged every 3–4 days using TrypLE™ Express (Life Technologies, (1×), no phenol red, Cat# 12604021, 3 min 37C incubator) for up to 10 passages. Note that P12 has normal mesenchymal look and morphology but manifested evidence of gradual loss of immunosuppressive ability. ADSCs were expanded and maintained in 5% Human Platelet Extract (Cook Regentec, Stemmulate, PL-SP-100) in DMEM supplemented with l-Glutamine and Pen/Strep.

### Generation of adipose-derived MSC constitutively expressing eGFP

RM20 adipose-derived stem cells at passage 0 were engineered to express eGFP under the control of the CMV promoter. A Lentiviral vector (VectorBuilder) containing eGFP was used to introduce eGFP for constitutive expression. 10,000 eGFP-positive cells were sorted at passage 1 and subsequently at passage 2 using the BioRAD S3 Cell Sorter. eGFP expression was confirmed by flow cytometry and fluorescence microscopy using the Keyence All-in-one Fluorescence Microscope BZ-X700 Series.

### PBMC assays

PBMC were isolated through standard Ficoll protocol (Ficoll-Paque Plus, GE Healthcare, cat# 95021-205) and co-cultured (100 μl) with ADSC (50 μl) plus minus vaccinia virus (50 μl) for 48 h on 96-well flat-bottom plates and in a total of 200 μl R10 medium (RPMI 1640 supplemented with 10% FBS, l-Glutamine and Pen/Strep). In some experiments the virus (50 μl) and stem cells (50 μl) were premixed and agitated on an orbital shaker at 37C (incubator) for 1 h, and then (100 μl of the mix) was added to the PBMCs without additional washing of any unbound virus. At the end of the 48 h incubation period the cells were recovered for staining and flow analysis directly or after an additional 4–5 h stimulation with K562 cells or PMA/Ionomycin (50 μl) with Monensin/Brefeldin A (50 μl) as needed.

### Flow cytometry analysis

Co-cultures of PBMC and stem cells were recovered by pipetting and transferred to V-bottom plates, where they were washed with FACS Buffer (1×PBS with 1% FBS) and surface stained for 30 min at 4C in FACS Buffer supplemented with the following antibody cocktail: anti-human CD3-PerCP/Cy5.5 (BioLegend, cat# 300328, at 1:50), anti-human CD335 or NKp46-PE (BioLegend, cat# 331908, at 1:50), anti-human CD69-APC (BioLegend, cat# 310910, at 1:50). The FACS buffer also contained a viability probe (ThermoFisher Scientific, LIVE/DEAD Fixable Violet Dead Cell Stain Kit, for 405 nm excitation, cat# L34964, at 1:1000). After staining the cells were washed twice with FACS Buffer, fixed in 2% PFA in 1×PBS for 15 min at RT, washed again with FACS Buffer to remove PFA and analyzed on BD FACSAria II. To evaluate cytotoxic functions in some experiments anti-human CD107a-AlexaFluor 488 (BioLegend, cat# 328610) was added directly to the co-cultures at 1:20 (10 μl/well) 5 h prior to recovery and surface staining followed by addition of Monensin at 1:1000 an hour later for additional 4 h incubation at 37 °C (BioLegend, cat# 420701-BL, 1000×).

### Intracellular stain

To evaluate IFNγ production in activated NK, NKT and T cells, in some experiments Brefeldin A was added at 1:1000 an hour after stimulation or 4 h prior to recovery and surface staining (BioLegend, cat# 420601-BL, 1000×). Monensin and Brefeldin A were added together when cells were to be evaluated for both IFNγ production and CD107a surface exposure. Following standard surface and viability staining cells were processed using the eBioscience Intracellular Staining Buffer Set (ThermoFisher, cat# 00-5523). Briefly, following surface staining with or without anti-CD107a-AlexaFluor 488, cells were fixed for 30 min with 1 part Fixation/Permeabilization Concentrate (cat# 00-5123) and 3 parts of Fixation/Permeabilization Diluent (cat# 00-5223), washed twice with 200 μl/well Permeabilization Buffer 10× (cat# 00-8333), diluted 1:10 in double distilled water), and stained with anti-human IFNγ-APC antibody (BioLegend, cat# 502512, at 1:50) in Permeabilization Buffer for 1 h at RT. Cells were washed twice in Permeabilization Buffer, fixed in 2% PFA in 1xPBS for 15 min at RT, washed again with FACS Buffer to remove PFA and analyzed on BD FACSAria II.

### NK cell immunosuppression

To evaluate the extent of ADSC-induced immunosuppression following the 48 h co-culture of PBMC with autologous or allogeneic ADSCs in the presence or absence of virus, the 250,000 PBMCs co-cultures were subjected to an additional brief 4 h stimulation at 37 °C (incubator) with 250,000 K562 (physiological stimulation of NK cells) or non-physiological stimulation with PMA/Ionomycin to assess the extent of NK cell viability/irreversible suppression. PMA (25 ng/ml final, Sigma, cat# P8139-5MG, diluted in DMSO to 5 mg/ml stock) and Ionomycin (1 μg/ml final, Sigma, cat# I-0634-1MG, diluted in DMSO to 1 mg/ml stock) were added as 4× solutions in medium. To evaluate cytotoxic activity of NK cells, the PBMC co-cultures were also stimulated in the presence of anti-human CD107a antibody in combination with Monensin/Brefeldin A as described above. Note that CD69+ surface expression was evaluated in duplicate wells in the absence of Monensin/Brefeldin A treatment due to severe interference.

### Plaque assay

Virus containing samples were stored at − 80 °C and subjected to a three-fold freeze (− 80 °C)/thaw (+ 37  C) cycle followed by sonication on ice-cold water for three 1 min intervals one min apart. Sonicated samples were serially diluted in vaccinia virus infection medium (DMEM supplemented with 2% FBS, l-Glutamine, Penicillin/Streptomycin). Plaque assays were performed in 24-well plates in duplicate wells. Briefly 200,000 CV1 cells were plated in 1 ml D10 medium per well overnight. Supernatants were aspirated and tenfold serial dilutions of the virus containing sample were applied to the CV-1 monolayer at 200 μl/well. Plates were incubated for 1 h at 37C (incubator) with manual shaking every 20 min. 1 ml CMC medium was layered gently on top of the cells and plates were incubated for 48 h. Plaques were counted after fixing the cells by toping the wells with Crystal Violet solution (1.3% Crystal violet (Sigma-Aldrich, C6158), 5% Ethanol (Pure Ethanol, Molecular Biology Grade, VWR, 71006-012), 30% Formaldehyde (37% v/v formaldehyde, Fisher, cat # F79-9), and double distilled water) for 3–5 h at room temperature, followed by washing the plates in tap water and drying overnight. CMC overlay medium was prepared by autoclaving 15 g Carboxymethylcellulose sodium salt (Sigma-Aldrich, C4888) and re-suspending with overnight stirring at RT in 1 L DMEM, supplemented with Pen/Strep, l-Glutamine, and 5% FBS, short-term storage at 4 °C.

### MTT viability assay

MTT assays were performed as previously described. Briefly, MTT (ThermoFisher, cat# M-6494, 5 mg/mL stock in 1×PBS, kept at− 20 °C) was added to cells (10 μl to 100 μl cells/well) on 96-well flat-bottom plates at a final concentration of 5 μg/mL and incubated for 1–2 h at 37 °C (incubator). Following incubation, cells were lysed by adding 100 μl of Isopropanol: 1 M HCl (24:1, supplemented with 10% Triton ×100, Sigma-Aldrich, ×100-100ML) and vigorous pipetting to dissolve the Formazan. Plates were read on Tecan InfiniteR 200 Pro and the MTT signal was measured within 1 h by subtracting OD at 650 nm from OD at 570 nm. Cells without MTT or Blank/Medium Alone wells were included as controls to eliminate background signals.

### Microscopy

Time course microscopic observations of virus infection were done on a Keyence All-in-one Fluorescence Microscope BZ-X700 Series. ADSCs were engineered to express eGFP and were followed on the GFP channel (1 s exposure) while virus infection with the TurboFP635-engineered L14 virus was monitored on the TRITC channel (3 s exposure). Images at 4× or 10× magnification were collected and overlaid with bright field (phase contrast, 1/50 s exposure).

### HLA and KIR analysis

HLA and KIR/MIC typing analysis was done through NGS by ProImmune (Oxford, UK) and Scisco Genetics (Seattle, USA), respectively. The presence/absence of the known KIR ligands A3/A11 (HLA-A), Bw4 (HLA-B) and C1/C2 (HLA-C) epitopes in the HLA alleles of our PBMC and ADSC donors was taken from http://www.dorak.info/mhc/nkcell.html. The − 21 M/T (Methionine/Threonine) dimorphism at the anchor amino acid from the leader sequence that predicts strong/weak binding and presentation of HLA-B-derived leader peptides by HLA-E, which provides inhibitory signaling though the NKG2A/CD94 receptors on NK cells, was taken from the Immuno Polymorphism Database (IPD) at http://www.ebi.ac.uk/ipd/.

### Data analysis and statistics

Data was plotted and analyzed for statistical significance using licensed Graph Prism software. Statistical significance was evaluated with the two-tailed Student’s T-test, p < 0.05, and statistically significant correlation was shown with the Pearson coefficient and corresponding p values.

## Results

### Adipose-derived stem cells provide potent amplification of vaccinia virus that can be restricted by the induction of IFN-mediated anti-viral state

**Fig. 1:**
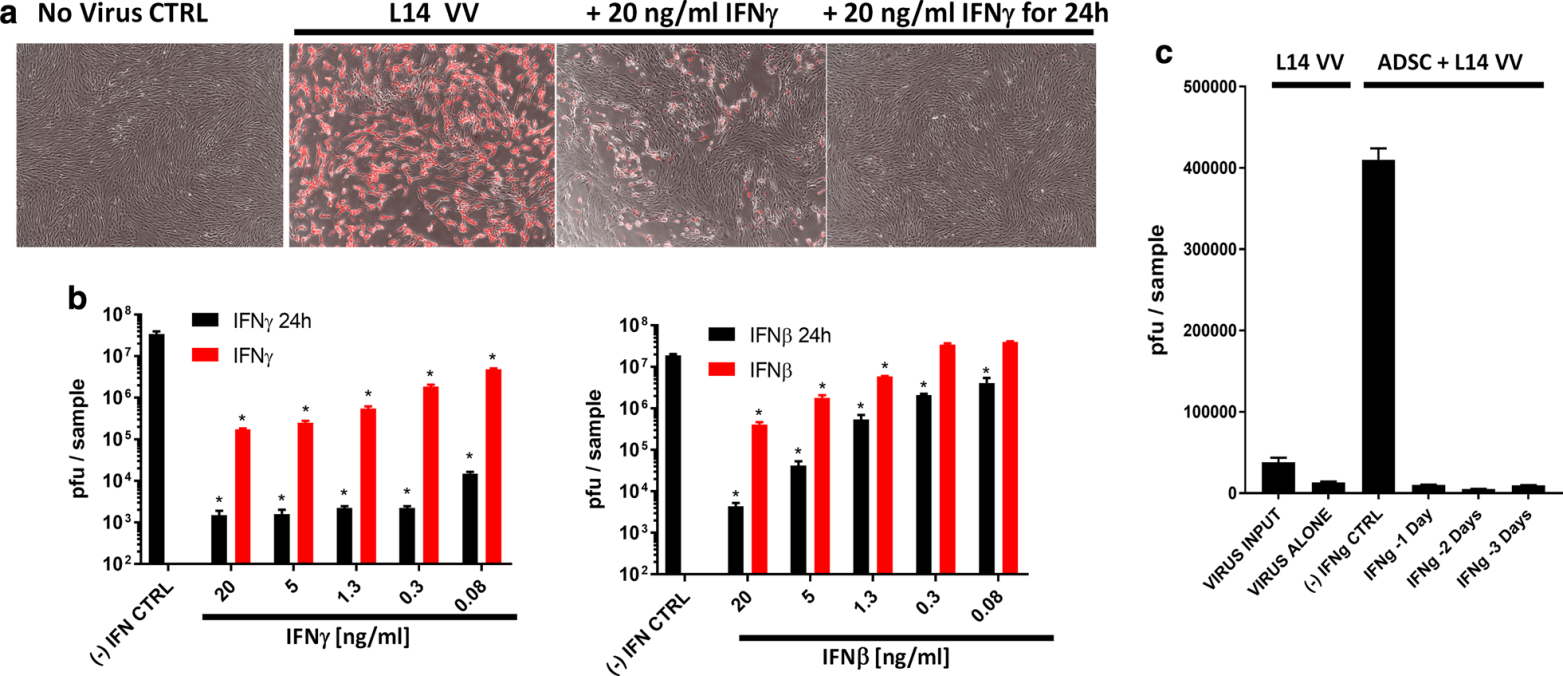
Adipose-derived stem cells provide potent amplification of vaccinia virus that can be restricted by the induction of IFN-mediated anti-viral state. **a**, **b** Both type I and II interferons protect ADSC against vaccinia virus (VV). 50,000 RM35 ADSC were infected in a 12-well plate with 10,000 pfu L14 VV, in the presence of increasing doses (0.08 to 20 ng/ml) of IFNγ or IFNβ added at the time of infection or 24 h earlier (IFNβ/γ 24 h). Fluorescence imaging (**a**) and plaque assays (**b**) at 48 h post infection show that a 24 h-pretreatment with both types of interferon is more effective at conferring protection. **c** Stability of the IFNγ-induced anti-viral state. 100,000 RM20-eGFP ADSC were infected in a 12-well plate with 100,000 pfu L14 VV and incubated for up to 4 days. The ADSC were either untreated, (−) IFNγ CTRL, or pre-treated with 20 ng/ml of IFNγ for 24 h administered 1, 2, or 3 days prior to virus infection. Plaque analysis shows significant virus amplification by the stem cells versus the dose administered, VIRUS INPUT, or remaining after co-culture in medium with no stem cells, VIRUS ALONE. The ability of the stem cells to amplify the virus was completely abrogated by interferon pretreatment as compared to the no interferon control group, (−) IFNγ CTRL. Bars represent duplicate measurements ± SD. Statistically significant differences (Student T-test, p < 0.05) based on duplicates versus (−) IFN CTRL are marked with asterisks

Tumor cells frequently harbor defects in type I interferon signaling that render them sensitive to oncolytic virus infection [59]. In addition to type I interferons, the type II IFNγ is also known for its critical role in restricting vaccinia virus infection in vivo through the induction of the so-called anti-viral state in both healthy and some interferon-responsive tumor cell lines [4]. Adipose-derived stem cells are normal untransformed mesenchymal stem cells that can be rapidly expanded ex vivo and can be used as a vehicle for delivery of oncolytic vaccinia virus. We investigated the potential of these cells to amplify vaccinia virus as well as to respond to the protective anti-viral effects of interferons. Here, we demonstrate that adipose-derived stem cells are highly permissive for vaccinia virus infection, showing amplification potential equivalent to levels observed in the highly permissive A549 human lung carcinoma cells. Both type I and II interferons protected stem cells against vaccinia virus infection (Fig. 1a, b, Additional file 1: Figure S1A), consistent with these cells being untransformed and having functional anti-viral interferon responses. Protection, however, was less efficient when interferon was given concurrently rather than 24 h prior to virus exposure. The combination of type I and II interferon didn’t further enhance protection, indicating the absence of a significant synergistic effect (Additional file 1: Figure S1B). Of note, the anti-viral state induced by interferon treatment was stable, lasting for several days after a transient 24 h exposure to IFNγ (Fig. 1c, Additional file 1: Figure S1C). Thus, type I and II interferon responses, while protecting the stem cells against virus infection and potentially improving their immunosuppressive abilities [51], can have the unfortunate effect of also compromising their ability to deliver and amplify vaccinia virus in vivo.

### ADSCs promote the oncolysis of resistant tumor cell lines through a combination of virus amplification, tumor cell recruitment and secretion of factors sensitizing the resistant tumor cells to virus infection

**Fig. 2:**
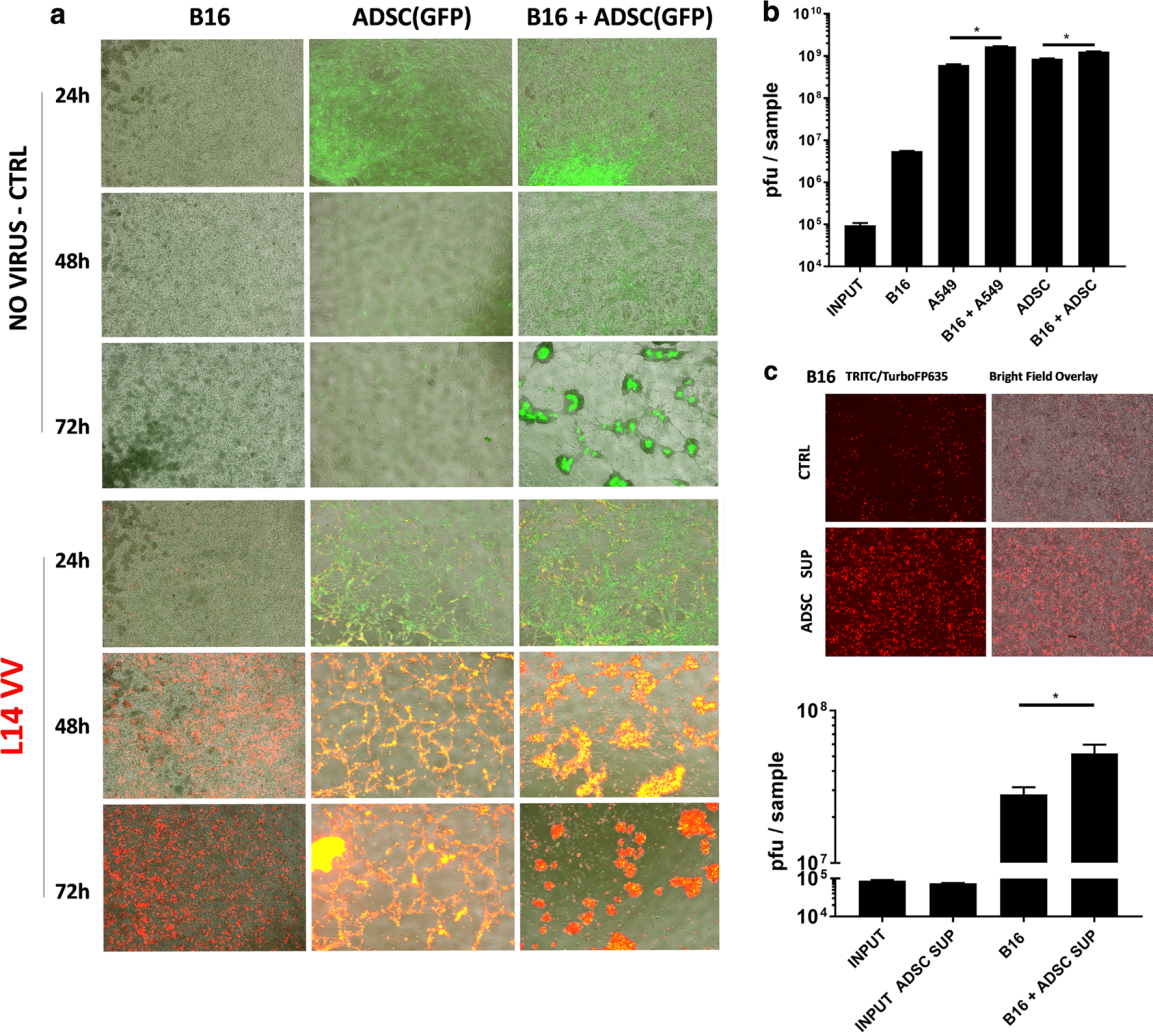
ADSCs promote the oncolysis of resistant tumor cell lines through a combination of virus amplification, tumor cell recruitment and secretion of factors sensitizing the resistant tumor cells to virus infection. **a** Human ADSC promote the oncolysis of resistant B16 melanoma cells through augmented amplification of the TurboFP635-engineered L14 vaccinia virus. The figure shows fluorescence image analysis of 1 × 10^6^ B16 cells cocultured with 2 × 10^5^ eGFP-labelled RM20 adipose-derived stem cells (×4 magnification) in a 12-well plate. B16 and stem cells were infected together with 1 × 10^5^ pfu virus (MOI = 0.1 to B16) and incubated for up to 72 h. **b** Plaque assay analysis of vaccinia virus amplification in the coculture experiment as described in **a** demonstrating that the viral titers recovered from the B16 + ADSC or B16 + A549 cocultures exceed the combined virus output from the individual cells infected in separation. The A549 lung carcinoma cells were used as a highly vaccinia virus permissive positive control. **c** ADSCs sensitize resistant tumor cells to virus infection. Supernatants from the human RM20 ADSC sensitize B16 melanoma to L14 vaccinia virus infection (×4 magnification). B16 cells were infected in triplicates as in **a** with L14 vaccinia virus at MOI of 0.1. The effect of supernatants on the infection of B16 cells with the L14 virus was analyzed using the TurboFP635 fluorescence (top panels) and quantitated by plaque assays at 72 h post infection (bottom). Bars represent duplicate measurements ± SD. Statistically significant differences (Student T-test, p < 0.05) based on duplicates or triplicates are marked with asterisks as indicated

Human tumors demonstrate varying sensitivity to vaccinia virus infection, with some tumors being more resistant than others. We specifically wanted to evaluate the effect of using ADSC to enhance the delivery of vaccinia virus in the context of resistant tumors. Stem cell delivery of oncolytic viruses can be used as a strategy to assist the oncolysis of resistant tumors by the introduction of highly sensitive cells that provide and sustain higher initial local virus production, thereby facilitating virus spread throughout the tumor. The murine B16 melanoma cells are known to be resistant to vaccinia virus infection and we tested whether in the presence of human adipose-derived stem cells the B16 cells can be targeted more effectively (Fig. 2a, Additional file 2: Figure S2A). Using eGFP-labeled ADSCs (green) to visualize and distinguish them from the unlabeled B16 cells (grey), we could observe that in confluent environments stem cells tended to cluster together while at the same time attracting the unlabeled melanoma cells. In the presence of the virus this attraction resulted in the formation of highly infected yellow stem cell clusters surrounded by intensively red-colored infected B16 cells. These effects were associated with a dramatically improved oncolysis of the monolayer of resistant murine B16 melanoma cells (Fig. 2a). Improved targeting of the resistant B16 cells was also observed with ADSC derived from another donor (Additional file 2: Figure S2B). Successful oncolysis, however, was compromised if the stem cells were pretreated with IFNγ (Additional file 2: Figure S2C) or were insufficient in numbers (Additional file 2: Figure S2D), suggesting that the observed antitumor potential was dependent on amplification of the virus by the stem cells. Our findings indicate that ADSCs have the unique property of both amplifying the virus (approx. 10,000-fold or 5000 pfu/cell) and spreading it to the tumor cells, which can be attributed to higher local multiplicity of infection (MOI) as well as some form of chemoattraction. Importantly, the viral titers recovered from these cocultures demonstrated that the viral output of the mixed stem and melanoma cells was greater than the combined outputs of the individually infected cells (Fig. 2b), suggesting that the highly permissive cancer or stem cells can sensitize the resistant melanoma cells to infection with vaccinia virus. We could further demonstrate that this effect is at least in part due to the secretion of still unidentified soluble factors present in the supernatants of ADSCs (Fig. 2c). Supernatants from different ADSCs donors could provide similar sensitization of both the murine B16 and the extremely resistant human K562 cancer cells (Additional file 2: Figure S2E), but the observed potentiating effects on the frequency of infected cells and virus amplification were relatively small (approximately twofold) and importantly insufficient for the eradication of these resistant cancer cells (Additional file 2: Figure S2F–H), indicating that successful therapy of resistant tumors might require both the sensitization and the amplification properties of the stem cells, as well as their ability to recruit murine and human tumor cells (Fig. 2a, Additional file 2: Figure S2H).

### ADSCs can be immunosuppressive towards NK cells in both autologous and allogeneic settings

**Fig. 3:**
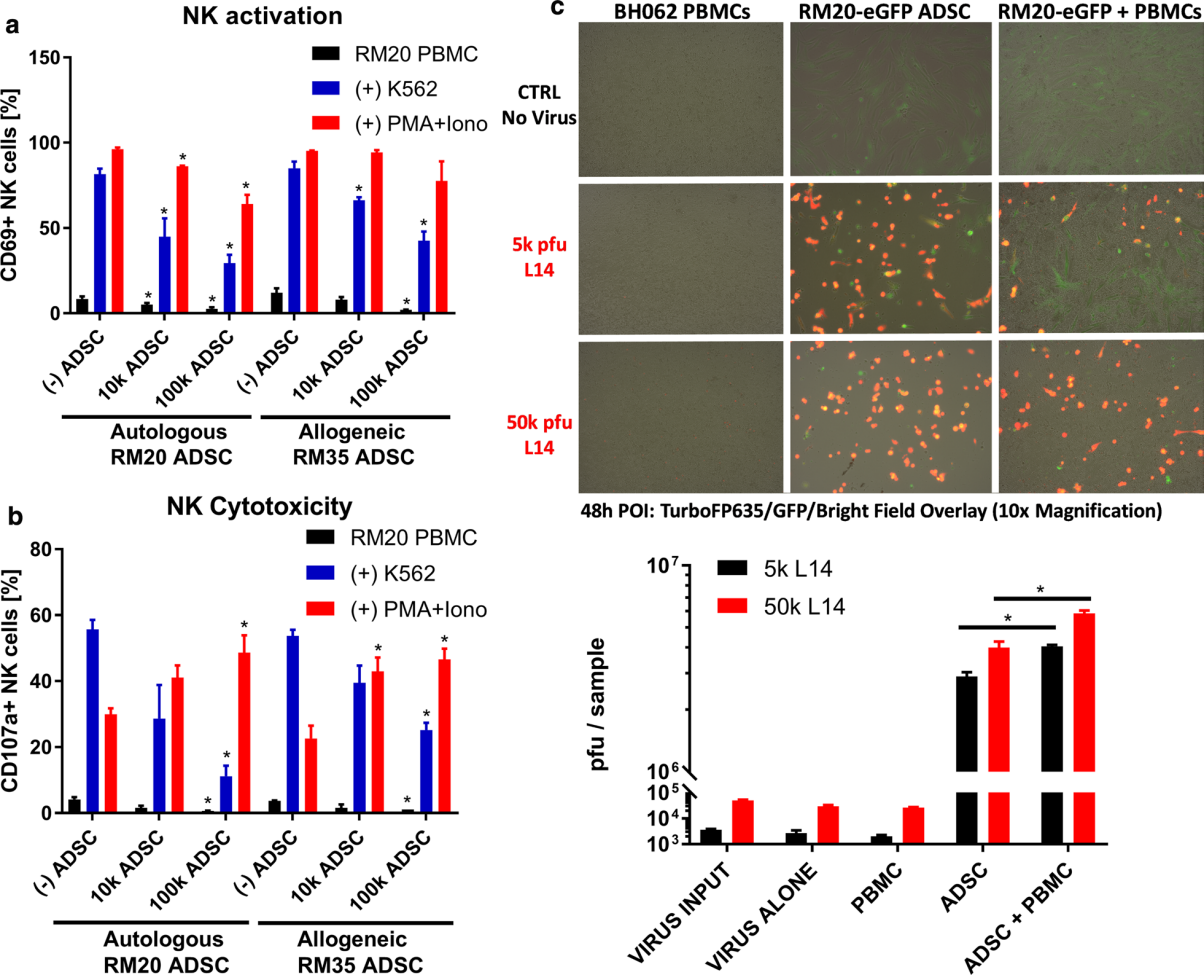
ADSC are suppressive against NK cells and can overcome allogeneic immune barriers. **a** ADSC suppress NK cells in autologous and allogeneic settings. Freshly isolated RM20 PBMCs (250,000) were cocultured for 48 h with 10, 000 or 100,000 autologous (RM20) or allogeneic (RM35) ADSC. To evaluate the extend of NK suppression the 48 h cocultures were subjected to an additional 4 h stimulation of NK cells with 250,000 K562 cells or PMA/Ionomycin. Data represent flow cytometry analysis of CD69 upregulation on gated live CD3-NKp46 + NK cells. **b** Flow cytometry analysis of NK cell cytotoxic functions using CD107a surface exposure as in **a**. Bars represent triplicate measurements ± SD. Statistically significant differences (Student T-test, p < 0.05) versus the corresponding (−) ADSC (PBMC alone) controls are marked with asterisks (**c**) ADSCs can amplify vaccinia virus in the presence of allogeneic PBMC. RM20-eGFP ADSCs (50,000) were infected with 5000 or 50,000 L14 VV alone or in the presence of 1 × 10^6^ allogeneic PBMC (BH062 blood donor) for up to 48 h. Overlay fluorescence imaging (top) and plaque assay (bottom) were used to evaluate vaccinia virus infection and amplification, respectively. Statistically significant differences (Student T-test, p < 0.05) based on duplicates ± SD are marked with asterisks

We hypothesized that the immunosuppressive potential of MSCs might be a key factor for their use as a Trojan horse delivery method for oncolytic viruses. It is frequently assumed that the immunosuppressive properties of MSCs would allow them to evade immune recognition and rejection even in highly unfavorable allogeneic settings [11]. To evaluate the potential of ADSCs to overcome allogeneic immune barriers and function as an effective Trojan horse, we tested their ability to immunosuppress and amplify vaccinia virus in the presence of allogeneic human PBMCs. MHC mismatches can typically trigger both NK and T cell responses, with NK cells representing the most significant initial barrier to both vaccinia virus and the Trojan horse, which is linked to their innate nature, high frequencies and ability to immediately respond to “missing self” due to allogeneic differences or vaccinia virus-induced MHC-downregulation, and without the need for T cell clonal expansion [58, 60]. Surface exposure and upregulation of CD69 and CD107a have been extensively used to study the immunosuppressive effects of MSCs on NK or T cell activation and cytotoxic functions, respectively [61–63]. Preliminary experiments with total PBMC, rather than purified immune subpopulations, indicated that a co-culture of at least 48 h was necessary for consistent detection of largely PMA/Ionomycin-reversible stem cell-mediated immunosuppression (Fig. 3a, b). This was a critical improvement over previous reports that were largely focused on the suppressive effects of MSC on purified NK and T cell populations using longer incubation times and exogenous cytokine support, thus introducing activation bias and ignoring the important role of innate and adaptive immune cell crosstalk [20, 39, 64, 65]. The co-culture with ADSCs demonstrated potent dose-dependent immunosuppressive properties against NK cells stimulated by brief exposure to the physiologically relevant K562 target cells in both autologous and allogeneic settings (Fig. 3a, b, Additional file 3: Figure S3A). Of note, in the co-culture settings the allogeneic stem cells failed to trigger any direct NK or T cell responses, which were decreased below the background levels of activation as measured in the PBMC alone controls (Fig. 3a, b, black bars). We were encouraged by our initial data confirming the immunosuppressive potential of ADSC in allogeneic settings but also wanted to explore the possibility that the actual potency of immunosuppression may be patient-specific and subject to certain allogeneic restrictions. Our optimized co-culture assay was specifically designed to reveal patient/MSC recipient-specific differences in both the immunosuppressive and virus amplification abilities of the stem cells.

### ADSCs can overcome allogeneic immune barriers and amplify VV even in the presence of some allogeneic PBMCs

We next evaluated the potential of ADSCs not only to immunosuppress but also to amplify vaccinia virus when co-cultured with PBMC from other individuals and against possible allogeneic immune barriers. We hypothesized that an unfavorable allogeneic PBMC environment might result in elimination or inactivation of the stem cells before they have a chance to amplify vaccinia virus. Surprisingly, the presence of allogeneic PBMCs didn’t interfere with virus amplification, suggesting that at least in some “permissive” cases the allogeneic stem cells remain “under the radar” and avoid immune recognition even under conditions of highly inflammatory virus infection (Fig. 3c). Moreover, in the presence of the allogeneic PBMCs the overall virus output was increased relative to the combined output of stem cells and PBMCs when infected in separation, suggesting that the stem cells can sensitize some subpopulations of normal PBMC to vaccinia virus infection in a way equivalent to their effect on tumor cells (Fig. 2b, c). These observations raised the relevant question as to whether successful and unrestrained virus amplification by the Trojan horse could be attributed to potent stem cell-mediated inhibition of anti-viral responses irrespective of any allogeneic differences or was it patient/MSC recipient-specific and limited to cases of a particularly close MHC match, hence not necessarily applicable to a larger cohort of MHC-diverse recipients.

### The potential of allogeneic stem cells to overcome immune barriers correlates with their ability to suppress virus-induced T, NK and NKT cell responses

**Fig. 4:**
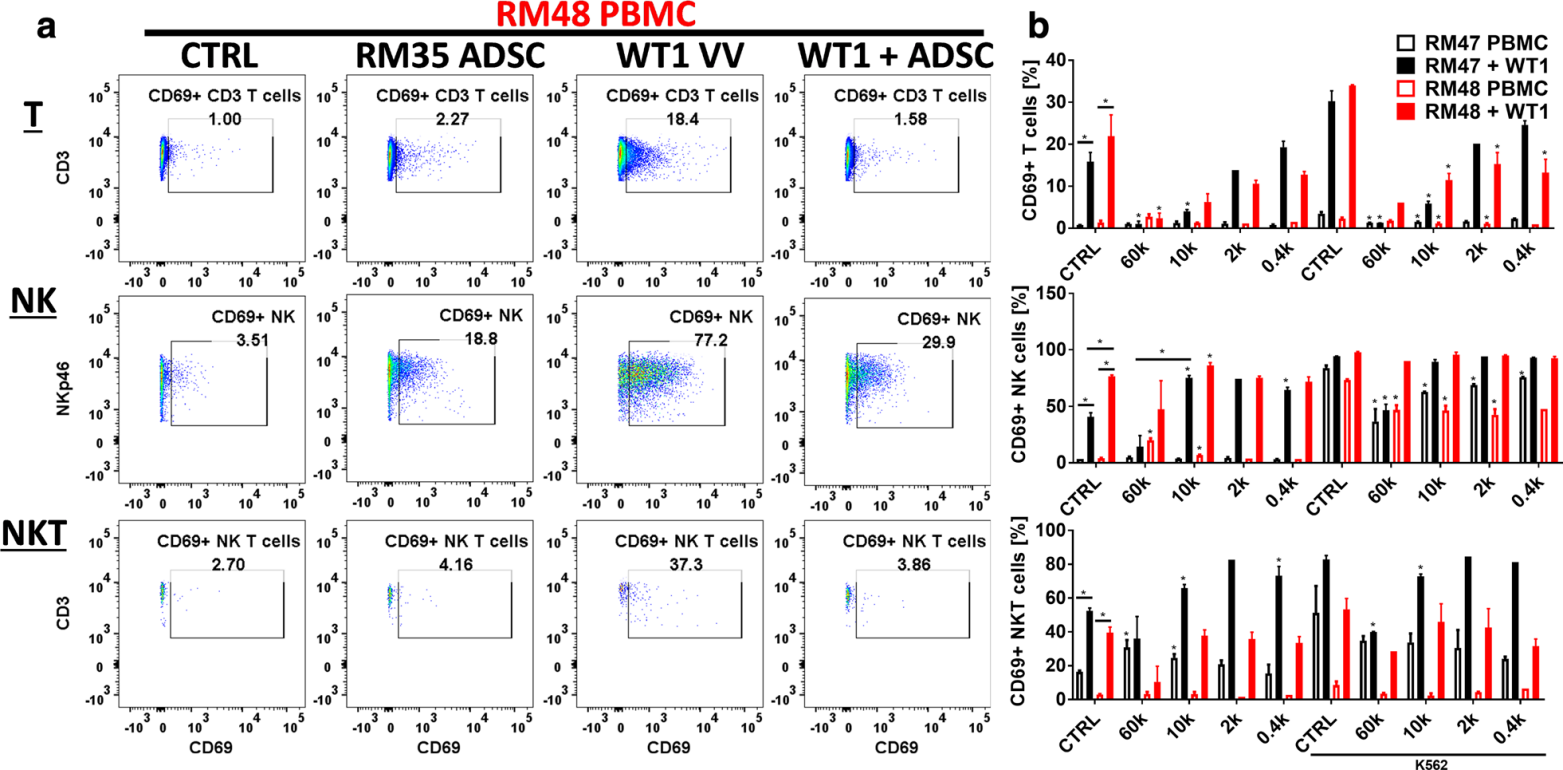
The potential of allogeneic stem cells to overcome immune barriers correlates with their ability to suppress virus-induced T, NK and NKT-like cell responses. **a** Allogeneic RM35 ADSC suppress vaccinia virus-induced innate and adaptive immune responses. 250 k PBMCs from 2 different blood donors (RM047 and RM048) were cocultured with 0.4–60 k allogeneic RM35 ADSC for 48 h in the presence or absence of 10 k pfu of WT1 VV. The figure shows a representative flow cytometry analysis of gated live T, NK, and NKT-like cells (see Additional file 4: Figure S4A) from blood donor RM048 at the highest 60 k ADSC dose. **b** Summary of the modulation of anti-viral responses by the RM35 ADSC in the PBMC donors RM047 and RM048 as in **a**. Bars show the percentage of CD69+ activated cells from each immune cell type as indicated. A brief 4 h stimulation with 250 k K562 cells at the end of the 48 h coculture period was used to evaluate the immunosuppressive potential of the allogeneic stem cells against NK cells. Bars represent duplicate measurements ± SD. Statistically significant differences (Student T-test, p < 0.05) versus the corresponding PBMC alone controls (CTRL) or as indicated are marked with asterisks. In the K562 groups asterisks indicate statistically significant difference versus the corresponding K562 CTRL, which represents PBMC cocultured alone without ADSC, with or without virus (solid versus empty bars, respectively)

The ability of the Trojan horse to amplify vaccinia virus in immunocompetent recipients might be critically dependent on the potential of stem cells to successfully inhibit anti-viral innate and adaptive immune responses. We therefore tested the ability of allogeneic ADSC from the RM35 donor to specifically suppress virus-induced responses mediated by NK and T cells in a new cohort of two PBMC donors/MSC recipients and with the goal to reveal possible patient-specific restrictions (Fig. 4a, Additional file 4: Figure S4A). Relative to T cells, NK cells were indeed the major responding population with the combination of virus and low doses of stem cells resulting in activation of more than 80% of the NK cells (Fig. 4b, Additional file 4: Figure S4B). Lower doses of the stem cells were insufficient for immunosuppression and unexpectedly increased virus-induced immune responses, possibly reflecting significantly augmented virus amplification. Higher doses of ADSCs, however, provided potent suppression of the weaker vaccinia virus-induced T cell responses in a dose-dependent fashion, indicating that stem cell-mediated immunosuppression overcomes the immune-stimulatory effect of augmented virus amplification. Suppression of anti-viral NK cell responses was evident only at the highest stem cell doses, and was not consistent across the two allogeneic blood donors tested. Interestingly, the NK cells from one of the blood donors responded directly to the allogeneic ADSCs (RM48, red empty bars). Using a standard 4 h K562 NK stimulation assay at the end of the 48 h PBMC/ADSCs/VV co-culture experiment, we demonstrate that the inconsistent suppression of the anti-viral NK responses might correlate with loss of the stem cells’ immune-privileged status and immunosuppressive abilities (Fig. 4b, NK panel, solid red/black bars versus K562 CTRL). We also identified a NKp46 + CD3 + NKT-like population of cells that responded vigorously to virus infection with upregulation of activation markers. This was also the only population of cells that manifested ability for rapid and selective expansion in response to vaccinia virus (Additional file 4: Figure S4B), consistent with the already established role of NKT cells in the control of virus infections [66].

### The potential of allogeneic stem cells to function as a Trojan horse is restricted by patient-specific differences suggesting that proper matching will be required

**Fig. 5:**
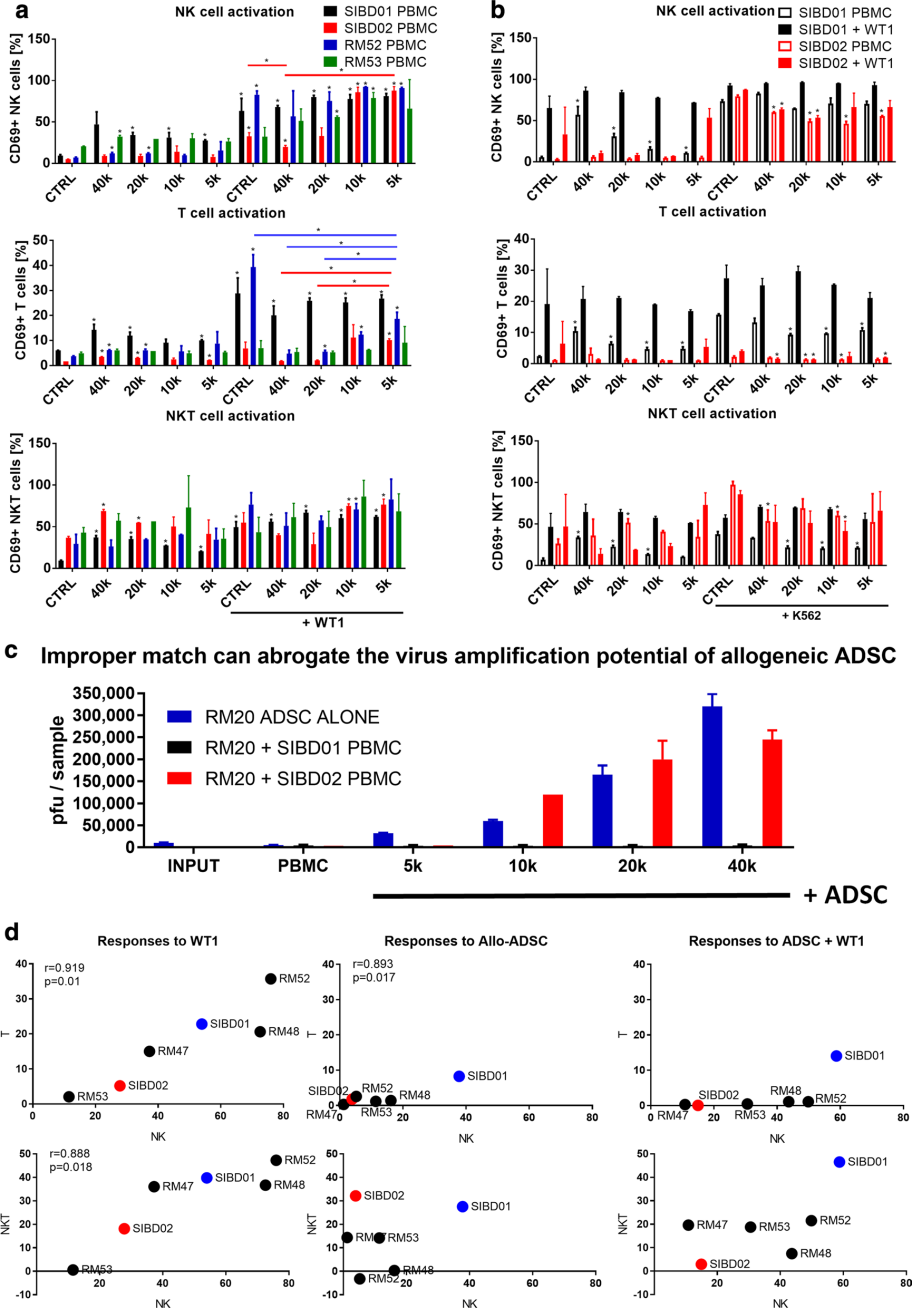
The potential of allogeneic stem cells to function as a Trojan horse is restricted by patient-specific differences suggesting that proper matching will be required. **a** PBMC donors demonstrate highly variable responses to the allogeneic stem cells and the virus alone or in combination. Flow cytometry analysis of gated live NK, T, and NKT cells from 48 h cocultures of 250 k PBMCs from 4 different blood donors with 5 k-40 k allogeneic RM20 ADSC, and in the presence or absence of 5 k pfu of WT1 VV. Data show the percentage of CD69+ activated cells from each cell type. Bars represent duplicate measurements ± SD. Statistically significant differences (Student T-test, p < 0.05) versus corresponding PBMC without virus or stem cells controls (CTRL) or as indicated are marked with asterisks. **b** Comparison of the immunosuppressive potential of the allogenic RM20 ADSCs against the NK cells from two blood donors in which the stem cells demonstrate differential ability to “stay under the radar”. Flow cytometry analysis of cocultures of PBMC from the SIBD01 and SIBD02 blood donors as in **a** followed by a 4 h stimulation with K562 cells to evaluate the extent of NK cell suppression. Bars represent duplicate measurements ± SD. Statistically significant differences (Student T-test, p < 0.05) versus the corresponding PBMC alone controls (CTRL) are marked with asterisks. In the K562 groups asterisks indicate statistically significant difference versus the corresponding K562 CTRL, which represents PBMC cocultured alone without ADSC, with or without virus (solid versus empty bars, respectively). **c** Plaque analysis of the 48 h cocultures as in **b** demonstrating that RM20 ADSC can amplify WT1 vaccinia virus only in the presence of allogeneic PBMC from the permissive SIBD02 but not the resistant SIBD01 blood donor. **d** Correlative analysis of NK, T, and NKT cell responses (% CD69 + normalized to untreated PBMC control) against the highest dose of the allogeneic ADSCs as in (**a**) and (Fig. 4b). Statistically significant correlations are indicated with corresponding p values and Pearson coefficients

Our next goal was to evaluate the immunosuppressive and virus amplification abilities of the other allogeneic ADSC derived from the RM20 donor and to test it in a larger cohort of blood donors/MSC-recipients to get a better understanding of the scope and magnitude of patient-specific restrictions. The low immunogenicity of stem cells has been associated with the secretion of various immunosuppressive factors as well as their extremely low level of MHC class I expression combined with the total absence of MHC class II or co-stimulatory molecules like CD80/86 or CD40. However, the potential of allogeneic stem cells to immunosuppress and “remain under the radar” [11, 67] can be significantly undermined by the progression of virus infection, which can not only wipe them out but also sensitize them to immune recognition by NK cells and T cells through Interferon-driven upregulation of MHC class I/II or surface expression of various infection-related stress molecules [24]. Thus, in the IFNγ-rich environments associated with vaccinia virus infection, the degree of matching between the allogeneic stem cells and the patient could control their ability to retain immunosuppressive properties and function as a Trojan horse. We validated our previous data (see Fig. 4b) showing that the same RM20 ADSC stem cells when tested against a panel of 4 new allogeneic PBMC donors can trigger direct NK, T, and NKT cell responses in a patient-specific manner (Fig. 5a, Additional file 5: Figure S5A). We observed a very high interpatient variability in the NK, NKT and T cell responses to both the allogeneic stem cells and to the virus. Moreover, the immunosuppressive properties of ADSCs appear to be failing in unfavorable allogeneic settings where the stem cells lose their immune privileged status and activate NK and T cells directly, even in the absence of the virus (Fig. 5b, Additional file 5: Figure S5B). We also demonstrate that these immunological differences have significant impact on the stem cells’ virus amplification potential. Importantly, improperly matched stem cells and blood donors representing potential MSC-recipients can completely abrogate the virus amplification potential of the allogeneic ADSC, thus revealing critical patient-specific differences that could lead to “permissiveness” or “resistance” to the Trojan horse (Fig. 5c). Additional correlative analysis of the immune responses against the virus revealed that vaccinia virus induces highly coordinated NK, NKT and T cell responses (Fig. 5d, Additional file 5: Figure S5C). Similar correlation was evident for the NK and T cells responses against the allogeneic stem cells, but responsiveness to virus versus ADSCs was discordant and likely independent of each other (Additional file 5: Figure S5D). Of note, we identified a pair of blood donors who were respectively highly resistant (SIBD01) and permissive (SIBD02) to one of our established allogeneic ADSC lines (RM20) and these extreme cases were used to further analyze the underlying mechanisms of patient-specific resistance to the Trojan Horse.

### Patient-specific immunological barriers regulate the survival of the Trojan horse and can limit the therapeutic potential of both genetically attenuated and wild type vaccinia virus strains

**Fig. 6:**
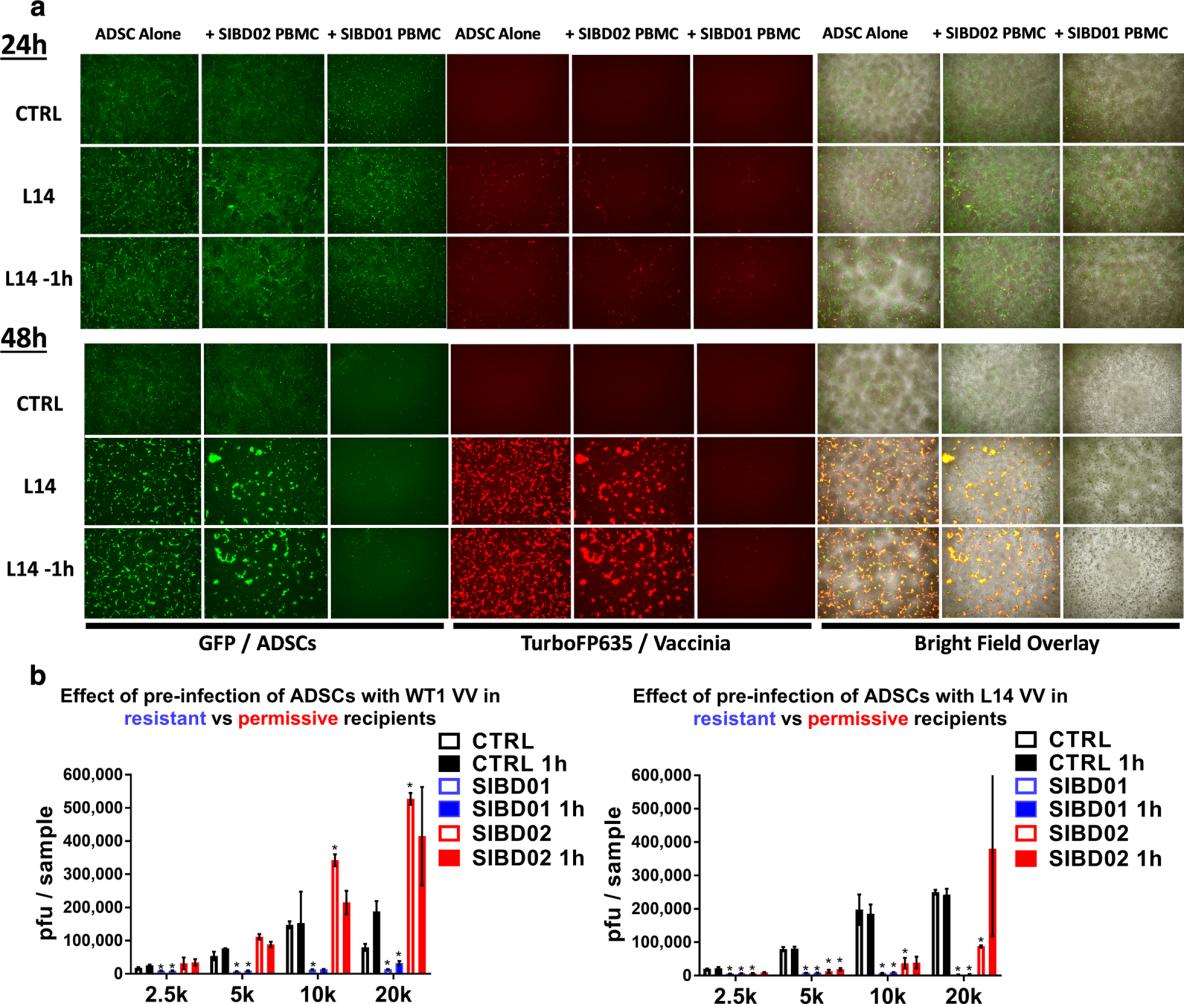
Patient-specific immunological barriers to the Trojan horse can limit the therapeutic potential of both genetically attenuated and wild type vaccinia virus strains. **a** Fluorescence imaging analysis of 20 k eGFP-labelled RM20 ADSC co-cultured with 20 k L14 virus (MOI of 1) for up to 48 h in the presence of 250 k PBMC from the resistant SIBD01 or permissive SIBD02 blood donors. Stem cells were infected with the virus at the time of coculture with the PBMC or were pre-infected for 1 h in 37 °C incubator with constant shaking and then added to the PBMC without washing away any unbound virus. **b** Plaque analysis of vaccinia virus amplification in 2–20 k ADSCs + PBMC cocultures as in **a**. Data represent means and SD based on independent duplicate wells and show comparison of parallel infection with the genetically attenuated L14 and wild type WT1 vaccinia virus. Bars represent duplicate measurements ± SD. Statistically significant differences (Student T-test, p < 0.05) versus same number of stem cells infected in the absence of allogeneic PBMC are indicated with asterisks

The total abrogation of the virus amplification potential of ADSC in certain allogeneic settings challenged their assumed immune privileged status and prompted us to investigate their survival in cocultures with allogeneic PBMC. We thought that immunological rejection could provide a possible explanation for the high resistance of some patients to an allogeneic Trojan horse. Monitoring stem cells’ survival in complex cocultures is limited due to their strong surface adhesion and tendency to cluster together, making it challenging to disaggregate them without losing viability. To visually evaluate the fate of allogeneic stem cells cocultured with PBMCs from resistant versus permissive donors and in the presence or absence of vaccinia virus infection, we utilized the eGFP-labelled RM20 ADSC line and the TurboFP635-engineered L14 vaccinia virus. Parallel viral titer analysis of the cocultures also included the WT1(ACAM2000) virus, which is a clonal isolate of wild type vaccinia virus that lacks the genetically attenuating elimination of the TK locus. This is the FDA-licensed USA stockpile of smallpox vaccinia virus vaccine that has been extensively used in humans with a well-established safety profile. Comparative analysis of the survival and amplification potential of the allogeneic Trojan horse in the case of permissive versus resistant recipient indicated that resistance was associated with the rapid loss of the stem cells in the presence of the resistant allogeneic PBMCs and in the absence of virus infection. These same stem cells remained intact when cocultured with PBMCs from the permissive recipient (Fig. 6a). To make sure that the allogeneic immunological barriers cannot be easily overcome by simply giving a head start to the virus and to more closely mimic its potential clinical application in the future, we also infected the Trojan horse an hour before exposure to the PBMCs, which was sufficient for approximately half of the virus to get into the cells (Additional file 6: Figure S6A). Despite potentially enhancing stem cell infection and accelerating virus amplification, the 1 h head start had a very small overall effect on the amplification potential of the Trojan horse (Fig. 6b). Interestingly, the permissive SIBD02 PBMCs partially suppressed the amplification of the L14 but not the WT1 virus. Contrary to the Lister-based Turbo-FP635-engineered L14 virus, which has the TK locus inactivated/removed as in most genetically attenuated vaccinia virus strains, WT1/ACAM2000 is a wild type TK-positive Wyeth vaccinia virus that has demonstrated higher amplification potential and ability to overcome stronger allogeneic barriers, consistent with possible faster amplification cycle or augmented ability to evade anti-viral immunity. These advantages of the WT1 virus were nevertheless insufficient to overcome the immunological barriers in the resistant recipient and were only marginally improved by initiating virus infection of the stem cells an hour prior to co-culture with the allogeneic PBMCs. Consequently, patient-specific resistance to the Trojan horse represents an important and therapeutically significant barrier to the use of cell-based delivery vehicles for oncolytic viruses like vaccinia and might be even more relevant for viruses that are genetically attenuated to improve safety or tumor selectivity. The important patient-specific differences observed in our studies raised the question of whether resistance to an allogeneic Trojan horse reflects a simple random MHC mismatching phenomenon between the donor stem cells and certain recipients or that some patients manifest broader patterns of permissiveness and resistance to multiple allogeneic stem cell lines, which can be associated with other intrinsic and more complex immunological characteristics.

### Patients’ resistance to the Trojan horse is associated with possible HLA mismatches and the rapid induction of anti-stem cell cytotoxic and interferon responses

**Fig. 7:**
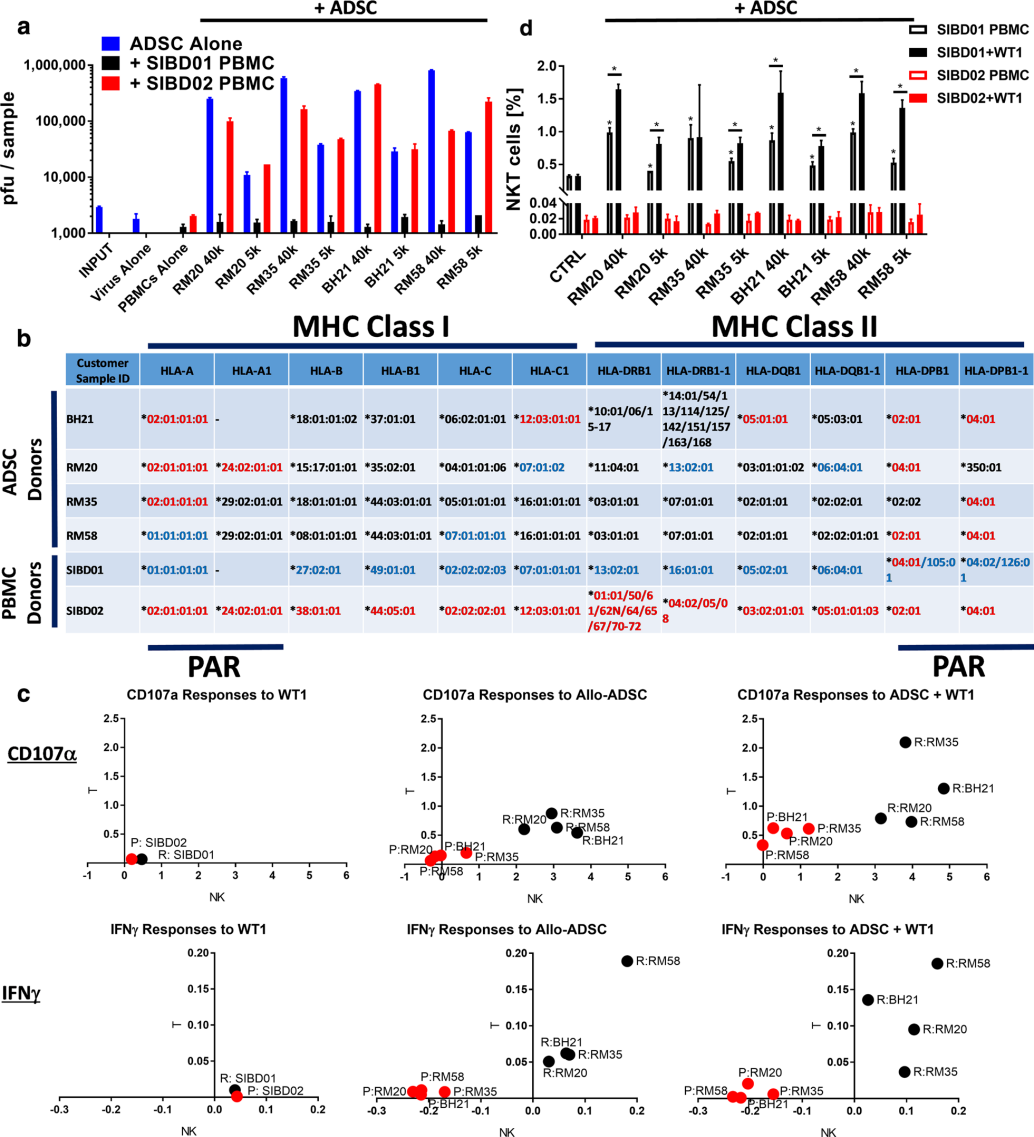
Patients’ resistance to the Trojan horse is associated with critical HLA mismatches and the rapid induction of anti-stem cell cytotoxic and interferon responses. **a** The SIBD01 and SIBD02 blood donors (MSC recipients) demonstrate broad resistance and permissivity to 4 allogeneic ADSC lines, respectively. Plaque assays were performed on 48 h cocultures of 250,000 PBMCs from the two blood donors with 40,000 or 5000 ADSCs infected with 5000 pfu of WT1 VV. The 4 allogeneic ADSC amplified vaccinia virus only in the presence of PBMC from the permissive SIBD02 blood donor. Bars represent duplicate measurements ± SD. **b** Precise HLA typing using NSG technology reveals loci potentially associated with permissiveness versus resistance to the four allogeneic ADSC lines, but alone cannot predict it (PAR permittivity associated region). To facilitate comparison the HLA alleles of the permissive and resistant blood donor are shown with red and blue font, respectively. **c** Correlative flow cytometry analysis of gated live NK and T cells from the PBMC/ADSC/WT1 co-cultures as in **a** showing that all 4 of the allogeneic stem cell lines tested induce much stronger CD107α and IFNγ responses in the NK and T cells from the resistant (black) but not permissive (red) blood donor, even in the absence of the virus. The figure shows the average percentages of CD107α or IFNγ single positive lymphocytes of each cell type based on triplicate wells and normalized to respective background (Untreated PBMC CTRL). Note that in the permissive blood donor the four allogeneic stem cells suppressed spontaneous NK cell-mediated IFNγ responses below background (Untreated PBMC CTRL). **d** CD3+ NKp46 + NKT-like cells are present at higher frequency in the resistant SIBD01 but not the permissive SIBD02 blood donor and expand in response to the allogeneic stem cell lines alone, which is increased even further in the presence of virus infection. Bars represent triplicate measurements ± SD. Asterisk was used to mark statistical significance (Student T-test, p < 0.05) relative to the respective (−) ADSC CTRL group or between groups as indicated

Comparative analysis of the resistant SIBD01 versus permissive SIBD02 blood donors tested against a panel of four available expanded allogeneic ADSCs indicated that they were broadly permissive and resistant, respectively (Fig. 7a). This broader permissiveness versus resistance correlated with partial matching mostly at the HLA-A and HLA-DP loci, with the broadly permissive donor having the most common HLA-A*02:01 allele, while the resistant one was HLA-A*01:01. HLA typing data alone are clearly insufficient to predict permissiveness versus resistance, as seen by the discordant case of an HLA-A*01:01 RM58 Trojan Horse (Fig. 7b). Rejection of closely HLA-matched cells can be alternatively explained by differences in the KIR Haplotype or in the balance of signaling through the NK cell inhibitory (KIR, NKG2A/CD94) or stimulatory (KIR, NKG2D) receptors. Indeed, our analysis shows that the discordant HLA-A*01:01 case could be linked to an important C2 KIR ligand mismatch (Additional file 7: Figure S7A) that has already been associated with insufficient KIR inhibitory signaling and NK cell-mediated rejection of HLA-Haploidentical iPSC [68]. Further mechanistic studies revealed that, regardless of the degree of partial HLA or KIR/KIR Ligand match, the ability of the Trojan horse to efficiently amplify the virus was associated with the absence of significant anti-stem cell IFNγ (Additional file 7: Figure S7B) and cytotoxic (Additional file 7: Figure S7C) NK and T cell responses across all the four allogeneic ADSC lines tested (Fig. 7c, Additional file 7: Figure S7D, E). Analysis of the PBMCs from the highly resistant SIBD01 recipient reveals that the improperly matched allogeneic stem cells induce a detectable IFNγ response even in the absence of virus infection that appears to originate from both NK and T cells. While T cells represent the bulk of early IFNγ-producing cells, NK cells demonstrate the highest proportional cytotoxic activity suggesting the existence of important cross talk between the innate and adaptive immune cell populations.

The PBMCs from the highly resistant SIBD01 donor also manifested a much higher frequency of CD3+ NKp46 + NKT-like cells relative to other blood donors tested, which could suggest an alternative explanation for the broader resistance to various allogeneic stem cell lines (Fig. 7d, Additional file 7: Figure S7F). The latter, however, were not found to be associated with significant IFNγ production or cytotoxic activity as measured by CD107a surface exposure at the 48 h coculture timepoint (Additional file 7: Figure S7B–D), but the potential involvement of NKT cells in the anti-viral response or the crosstalk between NK and T cells at earlier timepoints cannot be excluded. We specifically looked at anti-virus and anti-stem cell induced immune responses at earlier timepoints (Additional file 7: Figure S7G). Significant background immune cell activation was evident at 6 h that subsided by the 24 h timepoint. At 6 h only T and NKT cells from the resistant donor demonstrated stem cell-induced CD69 upregulation but not IFNγ responses. At 24 h all three subsets responded to the allogeneic stem cells by upregulation of the CD69 activation marker regardless of the virus. While the NK cell response was the most vigorous, only the NKT subset manifested statistically significant stem cell-induced IFNγ responses, suggesting that NKT cells might be the first immune cell subtype mounting effector cytokine responses against the allogeneic stem cells (Additional file 7: Figure S7G). The NK, NKT and T cells from the permissive donor didn’t upregulate CD69 or produced IFNγ in response to the stem cells at any timepoint. Of note, at the 24 h timepoint the allogeneic stem cells were even able to suppress IFNγ production by the permissive donor NK cells below background (Additional file 7: Figure S7G).

To study the potential detrimental effects of IFNγ secretion on the interactions between the immune cells and the allogeneic stem cells that are likely to occur in vivo, we performed experiments where the stem cells were pretreated with IFNγ prior to coculture with the PBMCs in the presence or absence of vaccinia virus (Additional file 7: Figure S7H–J). Contrary to our expectations that IFNγ-pretreatment would suppress immune responses due to inhibited virus amplification and improved immunosuppressive potential, as previously reported in the literature [53], the immune responses against the infected stem cells were further increased, specifically in the permissive donors suggesting that virus-associated infection and IFNγ-production might have the unfortunate effect of sensitizing immune cells to otherwise small and “under the radar” allogeneic differences resulting in compromised rather than improved immunosuppression of T and NK cells. Consistent with previous experiments (Figs. 4b, 5b), we also observed that the NK-specific stimulator cell line K562 induced indirect activation of NKT and T cells (Additional file 7: Figure S7J), providing further support to the notion of intensive NK-NKT-T cell crosstalk and interdependence. Such a crosstalk might also be critically important for the magnitude and kinetics of anti-viral or anti-allogeneic responses, thus playing a key role in the patient-specific restrictions to the Trojan horse.

Thus, while HLA or KIR matching might be informative and indeed play a significant role in determining the permissiveness versus resistance, the ultimate therapeutic efficacy of an allogeneic Trojan horse can be influenced by multiple other patient-specific differences including innate and adaptive immune cell composition as well as activation status or sensitivity to vaccinia virus/allogeneic mismatch. Such complex patient-specific differences would be much more properly evaluated by the development and application of robust companion diagnostic assays.

## Discussion

The goal of the work presented in the current manuscript was to evaluate the potential of using off-the-shelf allogeneic adipose-derived stem cells as a delivery vehicle to potentiate oncolytic virotherapy, leveraging the unique abilities of mesenchymal stem cells to amplify vaccinia virus and to transiently suppress anti-viral immunity. The immune system is known to play a dual role in oncolytic virotherapy of cancer. While the induction or potentiation of tumor-specific immunity is believed to play an important role in the long-term therapeutic efficacy of oncolytic viruses [69], the presence of innate and adaptive anti-viral immune barriers can significantly restrict the extent of direct vaccinia virus-mediated oncolysis. Importantly, recent data indicate that tumor burden might directly correlate with inadequate responsiveness to immunotherapy [70]. Limited oncolysis and the lack of immediate reduction in tumor burden can therefore compromise the immuno-stimulatory effects of oncolytic viruses and greatly reduce their therapeutic efficacy.

Improving direct oncolysis is contingent on designing effective strategies to overcome the existent immunological barriers to oncolytic viruses that include complement/antibody neutralization, the intrinsic tumor cell anti-viral interferon responses, as well as the elimination of infected cells by innate and adaptive immune cell populations such as NK cells and T cells. The NK cell responses appear to be of particular interest due to their innate characteristics and immediate nature, in sharp contrast to the time needed for expansion of virus-specific T cells [71, 72]. Of note, the latter branches of adaptive immunity appear to be critical for the ultimate clearance of the virus from the patient and warrant safety as well as therapeutic efficacy associated with the induction of tumor-specific immunity.

The therapeutic efficacy of oncolytic viruses is often restricted by the limited permissiveness of the tumor to virus infection and amplification [73], which reflects patient specific differences, including intrinsic tumor cell resistance [74], functional interferon anti-viral responses [4], or the presence of non-permissive tumor-associated stroma [75–77]. In tumors with relatively modest permissiveness, vaccinia virus might have insufficient time to efficiently colonize the tumor before it gets eliminated by the innate and adaptive anti-viral immune responses. Augmenting the oncolytic potential of vaccinia virus in such circumstances might therefore require a strategy that combines efficient delivery with a boosted local virus amplification that together could provide a favorable environment where virus spread and colonization occurs faster than the induction of protective anti-viral state.

Ex-vivo expanded autologous or allogeneic mesenchymal stem cells represent a unique delivery platform that offers the combined power of boosted local virus amplification and the ability to transiently suppress anti-viral innate and adaptive immunity, which is key to the success of oncolytic virotherapy in vivo. Given that the application of personalized autologous MSCs is an approach that is rather expensive and impractical for clinical development, the feasibility and limitations of using established and easily available allogeneic stem cell lines needs to be evaluated and investigated further. In the current study, we demonstrate that the impressive ability of mesenchymal stem cells to amplify vaccinia virus as well as to recruit and sensitize tumor cells to virus infection might significantly improve the therapeutic potential and broaden efficacy against resistant and low-permissive tumors. In addition, MSCs demonstrate the ability to effectively suppress anti-viral NK cell responses that represent the earliest and most significant innate immune barrier to tumor colonization and oncolysis [71, 72]. Of note, while MSCs appear to be immunosuppressive against NK cells in both autologous and allogeneic settings, as to date it has been shown in the literature, we demonstrate that their immunosuppressive ability and virus amplification potential remain subject to significant allogeneic barriers associated with the production of IFNγ and direct cytotoxicity by both NK and T cells. Such rapid and exaggerated allogeneic responses or the outright rejection of the stem cells, therefore, represent a significant obstacle to the use of off-the-shelf MSC- or alternative cell-based delivery platforms in the clinic.

The hypoimmunogenic and immunosuppressive characteristics of mesenchymal stem cells have justified their extensive use in the treatment of various autoimmune and inflammatory conditions, demonstrating similar persistence and equivalent therapeutic efficacy in both autologous and allogeneic settings. Conflicting reports demonstrate the superiority of using autologous stem cells, which probably reflects different disease background or requirements for brief immunosuppression versus long-term persistence and tissue repair [78]. In fact, numerous studies have already challenged the paradigm that allogeneic stem cells can be used in a one-size-fits-all universal donor setting, due to their hypoimmunogenic features. Instead, it becomes increasingly clear that MSC are not immune privileged and can induce allogeneic responses resulting in their ultimate rejection [79, 80].

Here, we argue that, to successfully utilize the potential of MSCs for the purposes of oncolytic virotherapy, it is necessary to build a much deeper understanding of the underlying mechanisms that control the balance between their immune evasive and immunogenic characteristics, and specifically how these are affected by the inflammatory environment associated with vaccinia virus infection. Importantly, long-term persistence of the allogeneic stem cells is irrelevant for the purposes of oncolytic virotherapy, while their short-term immunosuppressive and virus amplification potentials are essential and indispensable. Our demonstration that the combination of IFNγ production and direct cytotoxicity are associated with inability of the allogeneic Trojan horse to deliver and amplify vaccinia virus is critically important for the advancement of this therapeutic modality, as IFNγ plays a key role in both the control of vaccinia virus infection and the regulation of the immunosuppressive properties of MSCs. Multiple studies have demonstrated that exposure to IFNγ can significantly improve the immunosuppressive functions of MSCs by upregulation of IDO, iNOS/NO, COX2/PGE, and PD-L1 [50]. This would give stem cells the unique ability to support virus colonization by subverting IFNγ-driven immune activation and anti-viral immunity, but previous studies as well as our own findings indicate that IFNγ can also exacerbate small allogeneic differences and increase the immunogenicity of stem cells, potentially through up-regulated expression of MHC Class I/II or other costimulatory molecules. It should be emphasized that, while IFNγ-pre-treatment might be beneficial to enhance MSC-mediated immunosuppression for the treatment of autoimmune and inflammatory conditions, where long-term engraftment is unnecessary or unaffected by minor allogeneic differences, this is certainly not the case with oncolytic virus approaches that are critically dependent on the ability of the Trojan horse to amplify the virus. Accordingly, inappropriate and rapid IFNγ responses against the allogeneic stem cells might be sufficient to inactivate the Trojan horse, even in the absence of outright rejection and compromised persistence in vivo.

Our findings reveal that the responses to vaccinia virus and allogeneic stem cells alone or in combination are highly patient-specific, thus demonstrating the need for further mechanistic studies aimed to validate the relative contribution of IFNγ and direct cytotoxicity for the inactivation of the allogeneic Trojan horse. Understanding the basis for this patient-specific permissiveness versus resistance to the Trojan horse is challenging as it can reflect multiple sources of variability, including MHC I/II mismatches, differences in the MHC-binding Killer Cell Immunoglobulin-like receptors (KIR Haplotype or ligands) or differential frequency/activation status of innate immune cell populations such as NK or NKT cells. The use of allogeneic stem cells for oncolytic virus delivery is greatly facilitated by the lack of requirement for long-term survival and engraftment of the cells, making this approach likely to work across insignificant MHC mismatch barriers and restricting patient-specific resistance to relatively small groups of patients, who can be excluded from clinical trials with the use of simple diagnostic assays, as the ones presented in this study. Alternatively, a more frequent or broader patient-specific resistance to allogeneic Trojan horses would require the establishment of allogeneic MSC banks and matching the patients to the available stem cell lines, based on MHC typing data or in vitro diagnostic assays, which take into account all of the complex immunological characteristics of patients and particularly those conferring rapid and exaggerated anti-viral/-stem cell responses able to significantly limit therapeutic efficacy in highly resistant individuals.

Given that amplification of the virus in the stem cells proceeds within hours to several days after infection, while it takes more than a week for the adaptive T cell responses to contain virus spread and oncolysis, it becomes evident that the kinetics of the anti-viral/stem cell responses within the first few days of treatment would be critical for the ability of the Trojan horse to have a significant therapeutic effect in vivo. Consequently, the crosstalk and interplay between different innate and adaptive branches of the immune system need to be further investigated as these might be directly associated with the speed and magnitude of the anti-viral/stem cell immunity. The combination of exaggerated innate immune mechanisms sensitizing adaptive immunity against the virus or the allogeneic stem cells can be particularly detrimental. We were very interested by the finding that a patient can be broadly resistant to several allogeneic stem cell lines and that this broad resistance was also associated with unusually high frequency of NKp46 + CD^3+^ NKT-like cells, which were also the only population that expanded in numbers in response to both the virus and the allogeneic stem cells. Unfortunately, our attempts to link this NKT-like population of cells with effector functions able to directly interfere with the Trojan horse were unsuccessful, as these cells didn’t manifest any significant contribution to IFNγ production or cytotoxic activity. NKT cells are known to play a role in the control of viral infections, but their involvement in vaccinia virus responses and immunity hasn’t been investigated fully yet. The fact that this population of cells is unlikely to be responsible for the direct rejection or inactivation of the Trojan horse does not eliminate the possibility that NKT or NKT-like cells are critically important for directing and accelerating coordinated NK and T cell responses against the stem cells or the virus. Detailed kinetic and mechanistic studies would be necessary to evaluate the possibility that innate immune cells like NKT cells provide early cytokine help sensitizing NK and T cell to the presence of the virus and/or potential allogeneic differences, as suggested by some of our preliminary data (Additional file 7: Figure S7D). The importance of the crosstalk between the innate (NK/NKT cells) and adaptive (B/T cells) arms of the immune system, as revealed by our data, suggests the existence of correlation between patient-specific NK and T cell responses. Despite the rather limited cohort of PBMC donors tested, it becomes evident that patients mount coordinated NK, NKT and T cell responses against the virus and allogeneic stem cells (Fig. 5d). On the other hand, the comparative responsiveness to the virus and stem cells appears discordant and highly patient-specific (Additional file 5: Figure S5C), which cannot be explained by HLA typing data alone and suggests the involvement of innate immunological differences that might not be the same with respect to the virus or possible MHC mismatches. These innate immunological differences can be associated with differences in the NK or NKT cells and more precisely with the balance of signaling through their activating versus inhibitory receptors such as NKG2A/NKG2C/NKG2D or KIR receptors, which like certain HLA alleles are highly patient-specific and have already been linked to resistance/susceptibility to infectious agents, autoimmune diseases, and cancer [81–86].

Overall, our data indicate that while autologous mesenchymal stem cells are potentially the optimal vehicles for the delivery and amplification of oncolytic viruses, the use of properly matched allogeneic stem cell lines in combination with robust companion diagnostic assays could provide a more practical and commercially viable alternative that guarantees consistent stem cell quality and validated amplification potential. This approach also provides the unique opportunity to utilize readily available off-the-shelf cell-based delivery platforms in a highly efficient personalized fashion.

## Conclusions

We demonstrate that mesenchymal stem cells, in addition to protecting naked viruses, can amplify vaccinia virus, enhance its delivery, and suppress anti-viral innate and adaptive immunity to potentiate oncolytic virotherapy. Our data also reveal that the ability of stems cells to amplify and deliver vaccinia virus is subject to allogeneic barriers that require proper patient-to-stem cell matching. We have also demonstrated that the resistance of patients to allogeneic carrier cells is associated with the induction of anti-stem cell IFNγ and cytotoxic responses, likely reflecting patient-specific HLA or KIR Haplotype mismatches. This study provides fundamental understanding of the molecular principles behind patient-specific resistance and will guide the future clinical development of cell-based delivery platforms for oncolytic virus therapy of cancer.

## Additional files

**Additional file 1: Figure S1.** Adipose-derived stem cells provide potent amplification of vaccinia virus that can be restricted by the induction of IFN-mediated anti-viral state. (A) Both type I and II interferons protect ADSC against VV. RM35 ADSC (50,000) were infected in a 12-well plate with 10,000 L14 VV, in the presence of increasing doses (ng/ml) of IFNγ or IFNβ added at the time of infection or 24 h earlier. Fluorescence imaging at 48 h post infection shows that pretreatment with both types of interferon is most effective at conferring protection. (B) The combination of type I and II interferon is not associated with synergistically enhanced protection against L14 VV. RM35 ADSC were pretreated for 24 h with IFNγ and IFNβ alone or in combination before infection with L14 VV as in Fig. 1a. The figure shows interferon-mediated suppression of virus amplification versus no interferon control group (CTRL). (C) RM20-eGFP ADSC (100,000) were infected in a 12-well plate with 100,000 L14 VV and incubated for up to 4 days. Stem cells were either untreated or pre-treated with 20 ng/ml of IFNγ for 24 h administered 1, 2, or 3 days prior to virus infection. The panels show a time course florescence image analysis of uninfected (eGFP+/GREEN) and infected dead (TurboFP635/RED) and infected live (YELLOW)) stem cells visualizing progression of virus infection.

**Additional file 2: Figure S2.** ADSCs promote the oncolysis of resistant tumor cell lines through a combination of virus amplification, tumor cell recruitment and secretion of factors sensitizing the resistant tumor cells to virus infection. (A) Human ADSC promote the oncolysis of resistant B16 melanoma cells through augmented amplification of the TurboFP635-engineered L14 vaccinia virus. The figure shows fluorescence image analysis of 1 × 10^6^ B16 cells cocultured with 2 × 10^5^ eGFP-labelled RM20 adipose-derived stem cells (4× magnification) in a 12-well plate. B16 and stem cells were infected together with 1 × 10^5^ pfu virus (MOI = 0.1 to B16) and incubated for up to 72 h (data party shown in Fig. 2a). (B) Human RM35 ADSC can also promote the oncolysis of the resistant murine B16 melanoma cells in vitro. Fluorescence imaging analysis of 1 × 10^6^ B16 cells cocultured with 200,000 ADSC and infected with 100,000 pfu L14 VV for up to 4 days. (C) IFNγ pretreatment protects stem cells only in the presence of relatively resistant B16 but not the highly permissive ADSC and A549 cells. 200,000 RM20-eGFP cells (0.2 M) were pretreated with 20 ng/ml IFNγ for 24 h, cocultured with 200,000 (0.2 M) RM20 ADSC, A549 or B16 cells, and infected with the L14 virus as described in (Fig. 2a). Note that IFNγ pretreatment of the stem cells compromised the oncolysis of the B16 monolayer. (D) Insufficient number of stem cells (2% or lower) results in incomplete oncolysis of the B16 monolayer. B16 cells and RM20-eGFP cells were cocultured and infected with L14 as described in (Fig. 2A). To evaluate the role of stem cell number/dose, we compared the oncolysis of the B16 monolayer in the presence of 200,000 (0.2 M) and 20,000 (0.02 M) stem cells. (E) Fluorescence imaging analysis of B16 (10,000) and K562 (100,000) cells infected with L14 virus at MOI of 0.1 for 96 h in 96-well flat-bottom plates in the presence of ADSC supernatants from different stem cell donors as indicated. (F) Plaque assay analysis of L14 (top) and WT1 (medium) vaccinia virus amplification in B16 cells as in (E) and MTT assay showing the absence of significant impact of ADSC supernatants alone on the survival of the infected B16 cells (Bottom). (G) Flow cytometry analysis of ADSC supernatant-potentiated infection of K562 cells as evidenced by slight increases in the frequency of infected cells, TurboFP635 + MFI, and viral titers, but lack of a significant effect on the overall survival of the highly resistant K562 cells, as measured by the MTT assay. (H) K562 cells were infected with L14 VV at MOI of 0.1 as in (E) but instead of supernatants K562 cells were cocultured with 5000 or 20,000 RM20-eGFP ADSCs in triplicates. Fluorescence imaging and flow cytometry analysis were used to show that the green fluorescent stem cells attract the unlabeled/grey K562 cells and dramatically increase the percentage of infected eGFP-negative TurboFP635 + K562 cells. Despite the potentiated infectivity of the highly resistant K562 cells, the stem cells ultimately fail to eradicate or significantly impact their overall survival, consistent with the minimal ability of these cells to amplify vaccinia virus, as shown in the NCI-60 human cell line screen previously. Statistically significant differences (Student T-test, p < 0.05) based on duplicates or triplicates versus control or as indicated are marked with asterisks.

**Additional file 3: Figure S3.** ADSC are suppressive against NK cells and can overcome allogeneic immune barriers. (A) ADSC-mediated immunosuppression does not affect the frequency of NK and T cells. Note that the PMA-Ionomycin treatment causes downregulation of the NKp46 marker used to identify and gate on NK cells, resulting in “disappearance” of the most activated NK cells.

**Additional file 4: Figure S4.** The potential of allogeneic stem cells to overcome immune barriers correlates with their ability to suppress virus-induced T, NK and NKT cell responses. (A) Gating strategy used to evaluate the effect of ADSC and vaccinia virus on activation of T, NK, and NKT-like cells as measured by upregulation of surface CD69 expression. (B) Summary of the modulation of the three immune cell populations as percentage of gated live lymphocytes in patients RM047 and RM048 (See Fig. 4b).

**Additional file 5: Figure S5.** The potential of allogeneic stem cells to function as a Trojan horse is restricted by patient-specific differences suggesting that proper matching would be required. (A) Patients demonstrate highly variable responses to the allogeneic stem cells and the virus alone or in combination. Flow cytometry analysis of gated live NK, T, and NKT cells from the 48 h cocultures of 250 k PBMCs from 4 different blood donors with 5–40 k allogeneic RM20 ADSC in the presence or absence of 5 k pfu of WT1 VV. Data show the percentage of each gated cell type in PBMC. (B) Flow cytometry analysis of cocultures of PBMC from the SIBD01 and SIBD02 blood donors as in (A) followed by a 4 h stimulation with K562 cells to evaluate the extent of NK cell suppression. (C) Correlative analysis of NK, T, and NKT cell responses (% CD69+ normalized to untreated control) against the WT1 virus, the allogeneic ADSCs or the combo as in F5A, partly shown in F5D. (D) Lack of correlation between NK, T and NKT responsiveness to the virus versus the allogeneic ADSC as in Fig. 5d. Statistically significant differences (Student T-test, p < 0.05) based on duplicates versus corresponding PBMC alone controls (CTRL) or as indicated are marked with asterisks.

**Additional file 6: Figure S6.** Patient-specific immunological barriers to the Trojan horse can limit the therapeutic potential of both genetically attenuated and wild type vaccinia virus strains. (A) Plaque analysis of supernatants after 1 h pre-incubation with ADSC in 37 °C incubator with constant shaking showing that at MOI of 1 (Ratio of VV to ADSC = 1) approximately half of the INPUT vaccinia virus gets attached or integrated in the pelleted cells and is absent from the supernatant. At higher MOI (fewer stem cells) most of the virus appears to remain free and requires longer time to integrate.

**Additional file 7: Figure S7.** Patients’ resistance to the Trojan horse is associated with critical HLA mismatches and the rapid induction of anti-stem cell cytotoxic and interferon responses. (A) The top table shows analysis of KIR Haplotypes as well as the presence of known KIR ligands including the Bw4 epitope (HLA-B) and the weak/strong C1/C2 epitopes (HLA-C). This table also includes analysis of the oligomorphic MICA/B molecules that serve as ligands for NKG2D activating receptors on NK cells. The bottom table shows the distribution and copy number of long(L)-inhibitory and short(S)-activating KIR receptors, with the total number of inhibitory and activating receptors present also summarized in the top table. Note the absence of clear correlation between permissiveness/resistance and KIR haplotype/KIR ligands, − 21 M/T dimorphism, and MICA/B oligomorphism. The RM58 stem cells manifest a potentially important KIR ligand C1/C2 mismatch with both the resistant SIBD01 and permissive SIBD02 blood donors, suggesting that such a mismatch alone is insufficient to confer resistance, which might also require additional and stronger HLA mismatching. (B–F) Flow cytometry analysis of gated live NK, NKT and T cells from the PBMC/ADSC/WT1 co-cultures, as in main Fig. 7, showing that all the 4 allogeneic stem cell lines tested induce much stronger CD107α and IFNγ responses in the NK and T cells from the resistant but not permissive blood donor even in the absence of the virus. The figure shows the average frequency and total numbers of IFNγ (B) or CD107α (C) single positive as well as the much lower-frequency IFNγ plus CD107α-double positive lymphocytes of each cell type. (E) Complete correlative analysis of gated live NK, NKT and T cells from the PBMC/ADSC/WT1 co-cultures as above (partly included in main Fig. 7c) showing the average percentages of CD107α or IFNγ single positive lymphocytes of each cell type based on triplicate wells and normalized to respective background (untreated controls). (F) Flow cytometry analysis as above showing that treatment with vaccinia virus or allogeneic stem cells doesn’t affect significantly the frequency of the gated lymphocyte populations, with the exception of the NKT-like cells from the resistant SIBD01 blood donor that expanded in response to the allogeneic stem cells alone and further in the presence of vaccinia virus infection. (G) NKT-like cells are the earliest produces of IFNγ. RM20 ADSCs (10,000 or 2000) were co-cultured with PBMC (250,000) from the resistant SIBD01 or permissive SIBD02 blood donors and WT1 VV (5000 pfu). Gated live NK, NKT and T cells were analyzed by flow cytometry for activation (CD69 surface stain) and effector functions (intracellular stain for IFNγ) at 6 h and 24 h timepoints. (H–J) Flow cytometry analysis of gated NK, NKT, and T cells from the PBMC of a resistant and several permissive blood donors co-cultured with allogeneic RM20 ADSCs untreated or pre-treated with 20 ng/ml IFNγ for 48 h. IFNγ pre-treatment enhances rather than suppresses NK and T cell responses in the permissive patients, in the presence (H) but also absence (I and J) of vaccinia virus. The data in (I) and (J) experiment also demonstrate that a later passage of the RM20 stem cells (p12) retains some T cell immunosuppression ability but loses ability to suppress NK cells. Stimulation of NK cells with K562 induces indirect T and NKT cell responses indicative of NK-NKT-T cell crosstalk. Data represent mean and SD of duplicate or triplicate wells showing % of gated population of cells or total number of cells. Asterisk was used to mark statistical significance (Student T-test, p < 0.05) relative to group control or between groups as indicated.

## Abbreviations

ADSC: adipose-derived stem cell
IFN: interferon
KIR: killer-cell immunoglobulin-like receptor
MHC: major histocompatibility complex
MSC: mesenchymal stem cell
PBMC: peripheral blood mononuclear cells
SVF: stromal vascular fraction
VV: vaccinia virus
