# Correction to: Delivery of oncolytic vaccinia virus by matched allogeneic stem cells overcomes critical innate and adaptive immune barriers

**DOI:** 10.1186/s12967-021-02866-7

**Published:** 2021-10-04

**Authors:** Dobrin D. Draganov, Antonio F. Santidrian, Ivelina Minev, Duong Nguyen, Mehmet Okyay Kilinc, Ivan Petrov, Anna Vyalkova, Elliot Lander, Mark Berman, Boris Minev, Aladar A. Szalay

**Affiliations:** 1 Calidi Biotherapeutics, San Diego, CA 92121 USA; 2 Institute of Biochemistry, Biocentre, University of Wuerzburg, Am Hubland, 97070 Würzburg, Germany; 3 Radiation Oncology, Moores Cancer Center, University of California San Diego, La Jolla, San Diego, CA 92037 USA; 4 California Stem Cell Treatment Center, Rancho Mirage, CA 92270 USA

## Correction to: J Transl Med (2019) 17:100 https://doi.org/10.1186/s12967-019-1829-z

The authors declare that a patent application entitled “Cell-based vehicles for potentiation of viral therapy” has been filed on August 8, 2019 by the inventors Dobrin Draganov and Aladar A. Szalay [1].
