# Supplementary figures and images for: Delivery of oncolytic vaccinia virus by matched allogeneic stem cells overcomes critical innate and adaptive immune barriers
Source: J Transl Med. 2019 Mar 27;17:100. doi: 10.1186/s12967-019-1829-z (PMC6437877; doi:10.1186/s12967-019-1829-z)

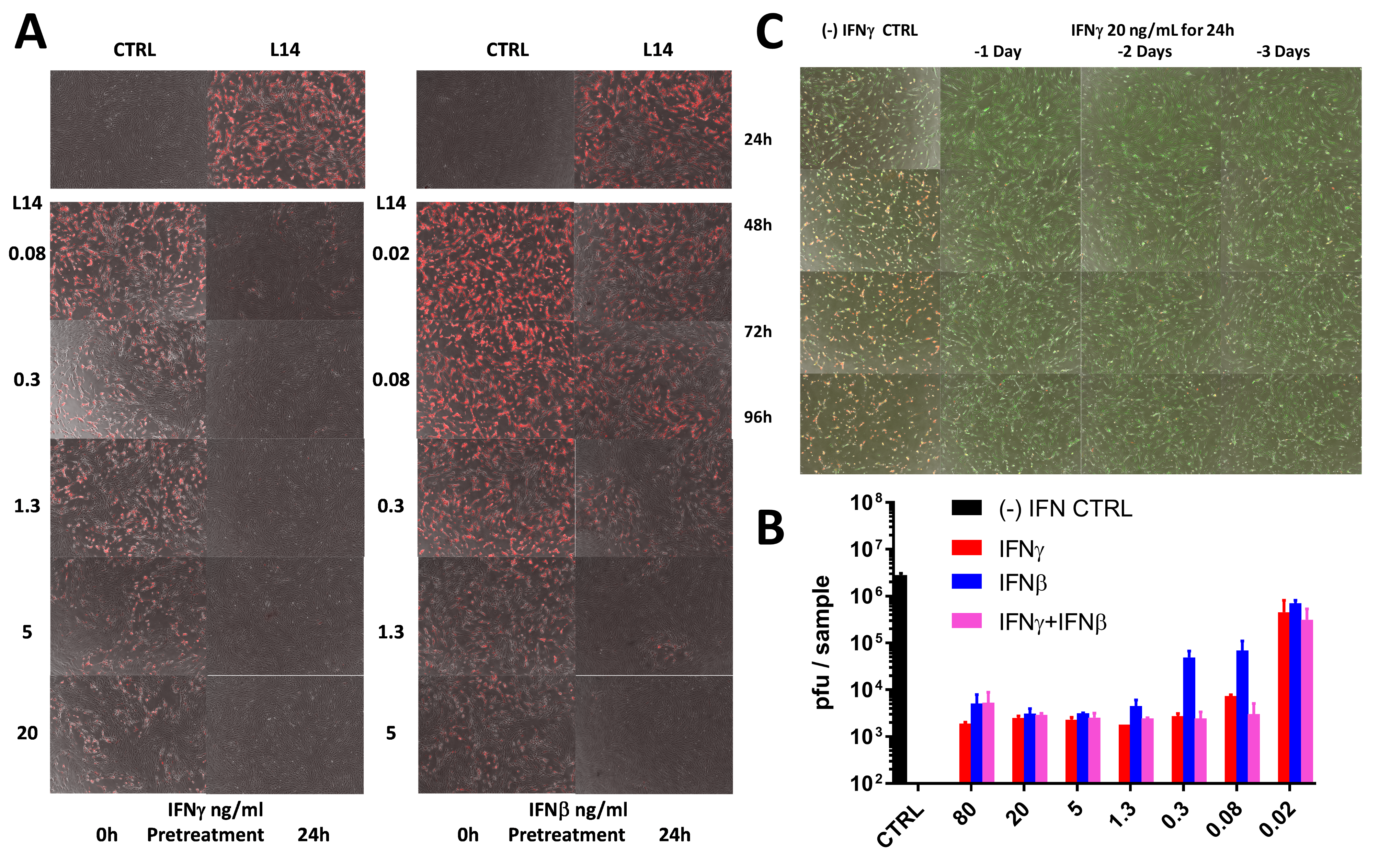

Supplement: Supplementary file 1 — Additional file 1: Figure S1. Adipose-derived stem cells provide potent amplification of vaccinia virus that can be restricted by the induction of IFN-mediated anti-viral state. (A) Both type I and II interferons protect ADSC against VV. RM35 ADSC (50,000) were infected in a 12-well plate with 10,000 L14 VV, in the presence of increasing doses (ng/ml) of IFNγ or IFNβ added at the time of infection or 24 h earlier. Fluorescence imaging at 48 h post infection shows that pretreatment with both types of interferon is most effective at conferring protection. (B) The combination of type I and II interferon is not associated with synergistically enhanced protection against L14 VV. RM35 ADSC were pretreated for 24 h with IFNγ and IFNβ alone or in combination before infection with L14 VV as in Fig. 1a. The figure shows interferon-mediated suppression of virus amplification versus no interferon control group (CTRL). (C) RM20-eGFP ADSC (100,000) were infected in a 12-well plate with 100,000 L14 VV and incubated for up to 4 days. Stem cells were either untreated or pre-treated with 20 ng/ml of IFNγ for 24 h administered 1, 2, or 3 days prior to virus infection. The panels show a time course florescence image analysis of uninfected (eGFP+/GREEN) and infected dead (TurboFP635/RED) and infected live (YELLOW)) stem cells visualizing progression of virus infection. [file 12967_2019_1829_MOESM1_ESM.tif]

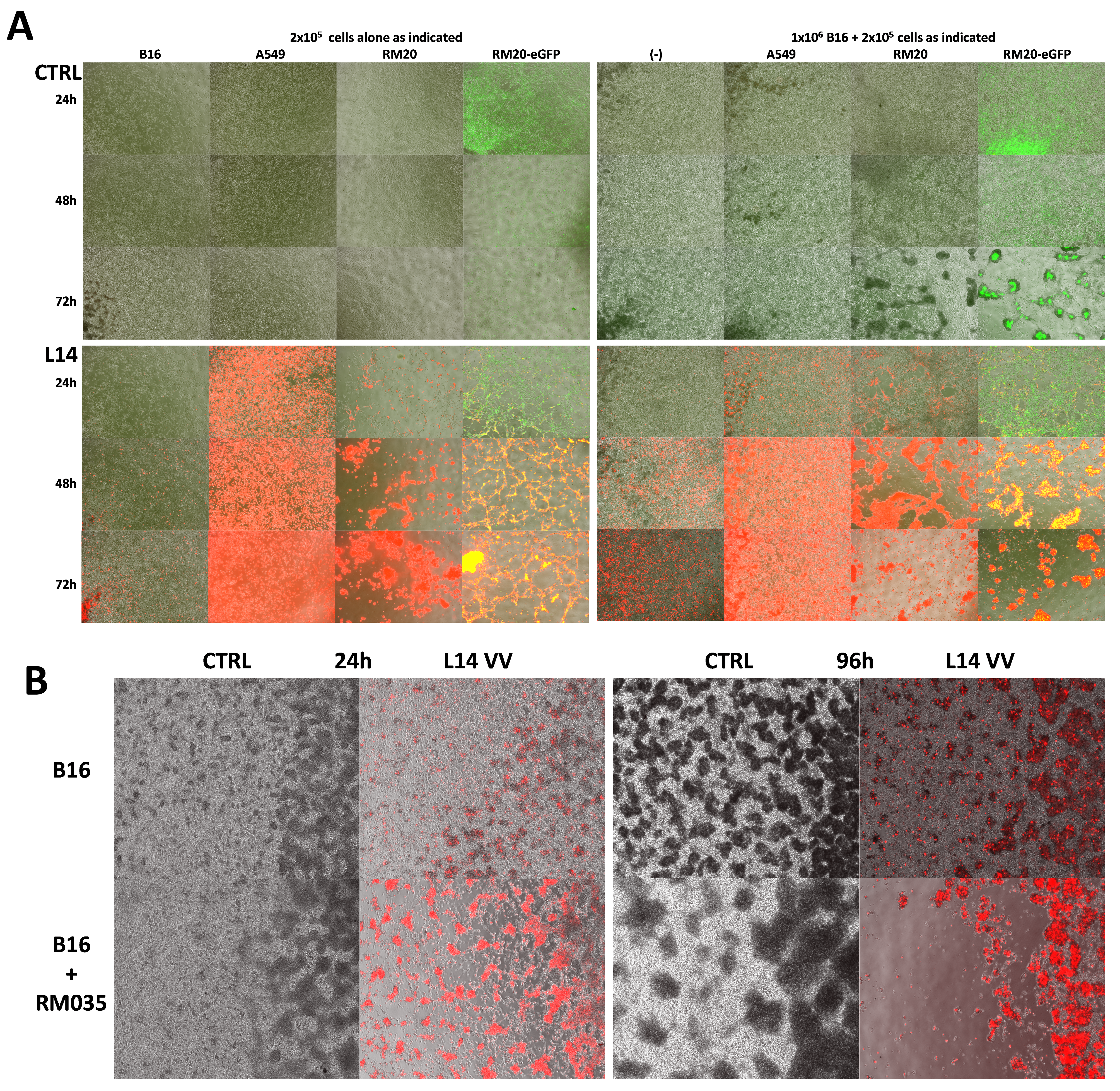

Supplement: Supplementary file 2 — Additional file 2: Figure S2. ADSCs promote the oncolysis of resistant tumor cell lines through a combination of virus amplification, tumor cell recruitment and secretion of factors sensitizing the resistant tumor cells to virus infection. (A) Human ADSC promote the oncolysis of resistant B16 melanoma cells through augmented amplification of the TurboFP635-engineered L14 vaccinia virus. The figure shows fluorescence image analysis of 1 × 10^6^ B16 cells cocultured with 2 × 10^5^ eGFP-labelled RM20 adipose-derived stem cells (4× magnification) in a 12-well plate. B16 and stem cells were infected together with 1 × 10^5^ pfu virus (MOI = 0.1 to B16) and incubated for up to 72 h (data party shown in Fig. 2a). (B) Human RM35 ADSC can also promote the oncolysis of the resistant murine B16 melanoma cells in vitro. Fluorescence imaging analysis of 1 × 10^6^ B16 cells cocultured with 200,000 ADSC and infected with 100,000 pfu L14 VV for up to 4 days. (C) IFNγ pretreatment protects stem cells only in the presence of relatively resistant B16 but not the highly permissive ADSC and A549 cells. 200,000 RM20-eGFP cells (0.2 M) were pretreated with 20 ng/ml IFNγ for 24 h, cocultured with 200,000 (0.2 M) RM20 ADSC, A549 or B16 cells, and infected with the L14 virus as described in (Fig. 2a). Note that IFNγ pretreatment of the stem cells compromised the oncolysis of the B16 monolayer. (D) Insufficient number of stem cells (2% or lower) results in incomplete oncolysis of the B16 monolayer. B16 cells and RM20-eGFP cells were cocultured and infected with L14 as described in (Fig. 2A). To evaluate the role of stem cell number/dose, we compared the oncolysis of the B16 monolayer in the presence of 200,000 (0.2 M) and 20,000 (0.02 M) stem cells. (E) Fluorescence imaging analysis of B16 (10,000) and K562 (100,000) cells infected with L14 virus at MOI of 0.1 for 96 h in 96-well flat-bottom plates in the presence of ADSC supernatants from different stem cell donors as indicated. (F) Plaque assay anal [file 12967_2019_1829_MOESM2_ESM.zip › SF2AB.TIF]

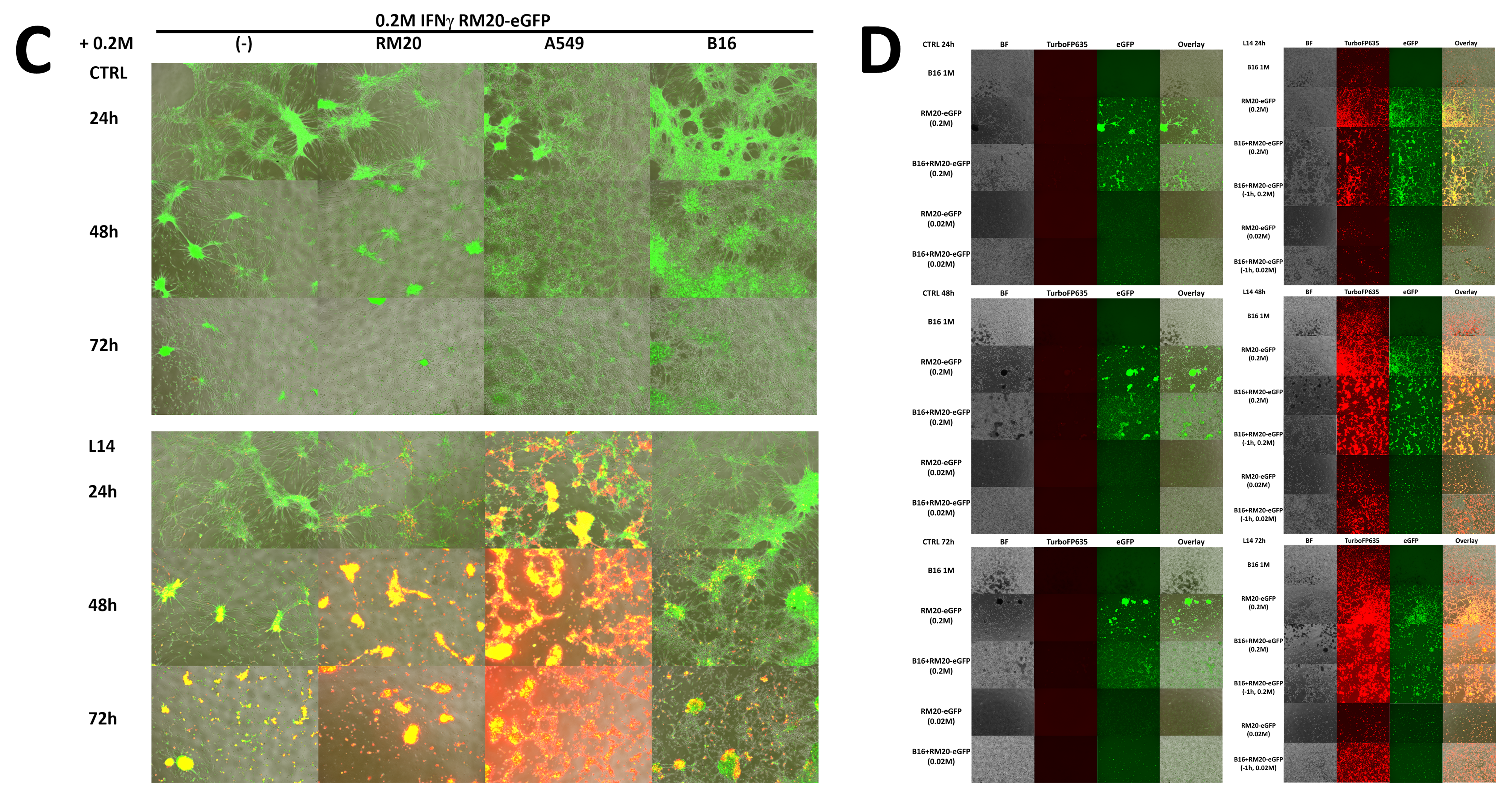

Supplement: Supplementary file 2 — Additional file 2: Figure S2. ADSCs promote the oncolysis of resistant tumor cell lines through a combination of virus amplification, tumor cell recruitment and secretion of factors sensitizing the resistant tumor cells to virus infection. (A) Human ADSC promote the oncolysis of resistant B16 melanoma cells through augmented amplification of the TurboFP635-engineered L14 vaccinia virus. The figure shows fluorescence image analysis of 1 × 10^6^ B16 cells cocultured with 2 × 10^5^ eGFP-labelled RM20 adipose-derived stem cells (4× magnification) in a 12-well plate. B16 and stem cells were infected together with 1 × 10^5^ pfu virus (MOI = 0.1 to B16) and incubated for up to 72 h (data party shown in Fig. 2a). (B) Human RM35 ADSC can also promote the oncolysis of the resistant murine B16 melanoma cells in vitro. Fluorescence imaging analysis of 1 × 10^6^ B16 cells cocultured with 200,000 ADSC and infected with 100,000 pfu L14 VV for up to 4 days. (C) IFNγ pretreatment protects stem cells only in the presence of relatively resistant B16 but not the highly permissive ADSC and A549 cells. 200,000 RM20-eGFP cells (0.2 M) were pretreated with 20 ng/ml IFNγ for 24 h, cocultured with 200,000 (0.2 M) RM20 ADSC, A549 or B16 cells, and infected with the L14 virus as described in (Fig. 2a). Note that IFNγ pretreatment of the stem cells compromised the oncolysis of the B16 monolayer. (D) Insufficient number of stem cells (2% or lower) results in incomplete oncolysis of the B16 monolayer. B16 cells and RM20-eGFP cells were cocultured and infected with L14 as described in (Fig. 2A). To evaluate the role of stem cell number/dose, we compared the oncolysis of the B16 monolayer in the presence of 200,000 (0.2 M) and 20,000 (0.02 M) stem cells. (E) Fluorescence imaging analysis of B16 (10,000) and K562 (100,000) cells infected with L14 virus at MOI of 0.1 for 96 h in 96-well flat-bottom plates in the presence of ADSC supernatants from different stem cell donors as indicated. (F) Plaque assay anal [file 12967_2019_1829_MOESM2_ESM.zip › SF2CD.TIF]

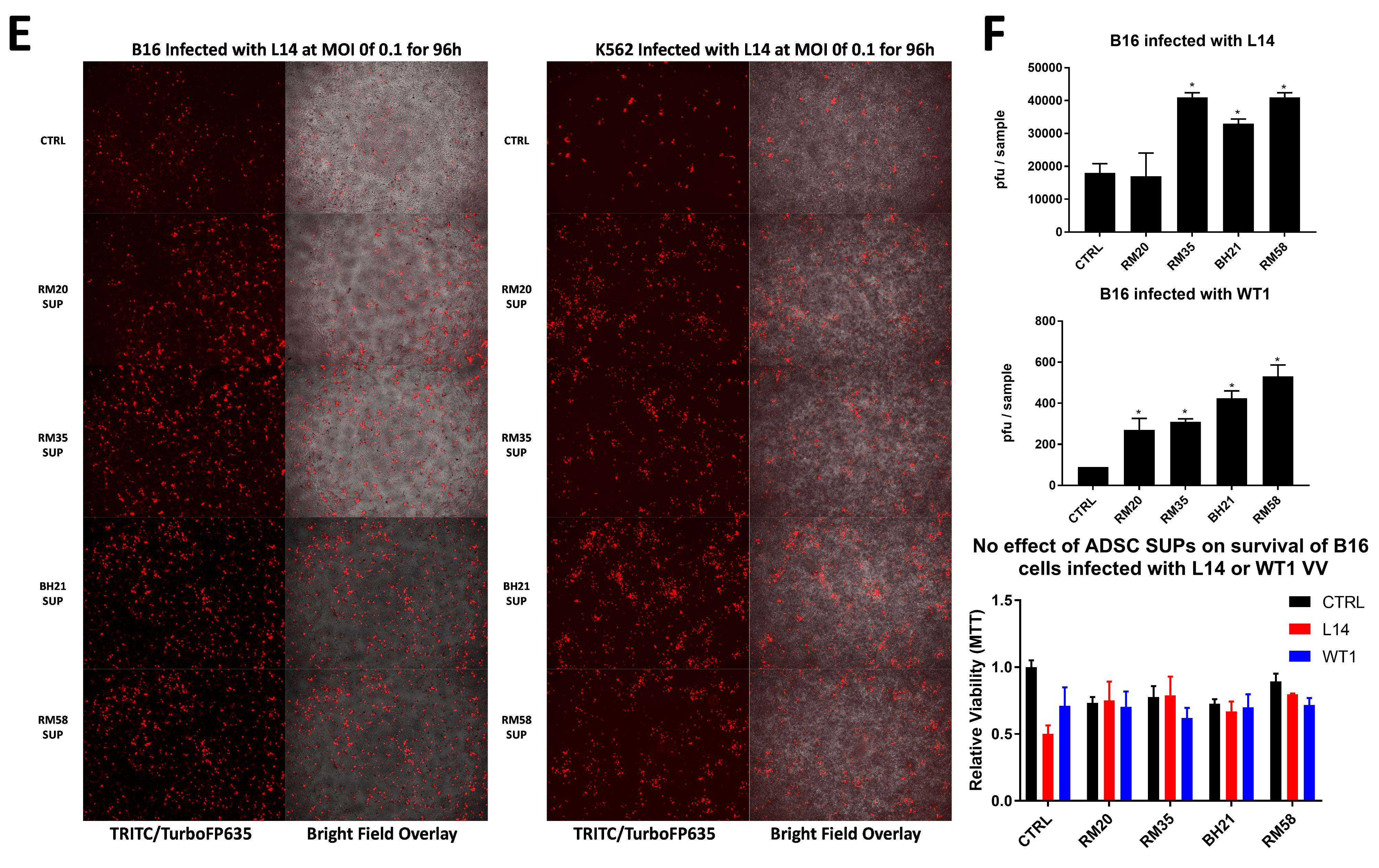

Supplement: Supplementary file 2 — Additional file 2: Figure S2. ADSCs promote the oncolysis of resistant tumor cell lines through a combination of virus amplification, tumor cell recruitment and secretion of factors sensitizing the resistant tumor cells to virus infection. (A) Human ADSC promote the oncolysis of resistant B16 melanoma cells through augmented amplification of the TurboFP635-engineered L14 vaccinia virus. The figure shows fluorescence image analysis of 1 × 10^6^ B16 cells cocultured with 2 × 10^5^ eGFP-labelled RM20 adipose-derived stem cells (4× magnification) in a 12-well plate. B16 and stem cells were infected together with 1 × 10^5^ pfu virus (MOI = 0.1 to B16) and incubated for up to 72 h (data party shown in Fig. 2a). (B) Human RM35 ADSC can also promote the oncolysis of the resistant murine B16 melanoma cells in vitro. Fluorescence imaging analysis of 1 × 10^6^ B16 cells cocultured with 200,000 ADSC and infected with 100,000 pfu L14 VV for up to 4 days. (C) IFNγ pretreatment protects stem cells only in the presence of relatively resistant B16 but not the highly permissive ADSC and A549 cells. 200,000 RM20-eGFP cells (0.2 M) were pretreated with 20 ng/ml IFNγ for 24 h, cocultured with 200,000 (0.2 M) RM20 ADSC, A549 or B16 cells, and infected with the L14 virus as described in (Fig. 2a). Note that IFNγ pretreatment of the stem cells compromised the oncolysis of the B16 monolayer. (D) Insufficient number of stem cells (2% or lower) results in incomplete oncolysis of the B16 monolayer. B16 cells and RM20-eGFP cells were cocultured and infected with L14 as described in (Fig. 2A). To evaluate the role of stem cell number/dose, we compared the oncolysis of the B16 monolayer in the presence of 200,000 (0.2 M) and 20,000 (0.02 M) stem cells. (E) Fluorescence imaging analysis of B16 (10,000) and K562 (100,000) cells infected with L14 virus at MOI of 0.1 for 96 h in 96-well flat-bottom plates in the presence of ADSC supernatants from different stem cell donors as indicated. (F) Plaque assay anal [file 12967_2019_1829_MOESM2_ESM.zip › SF2EF.TIF]

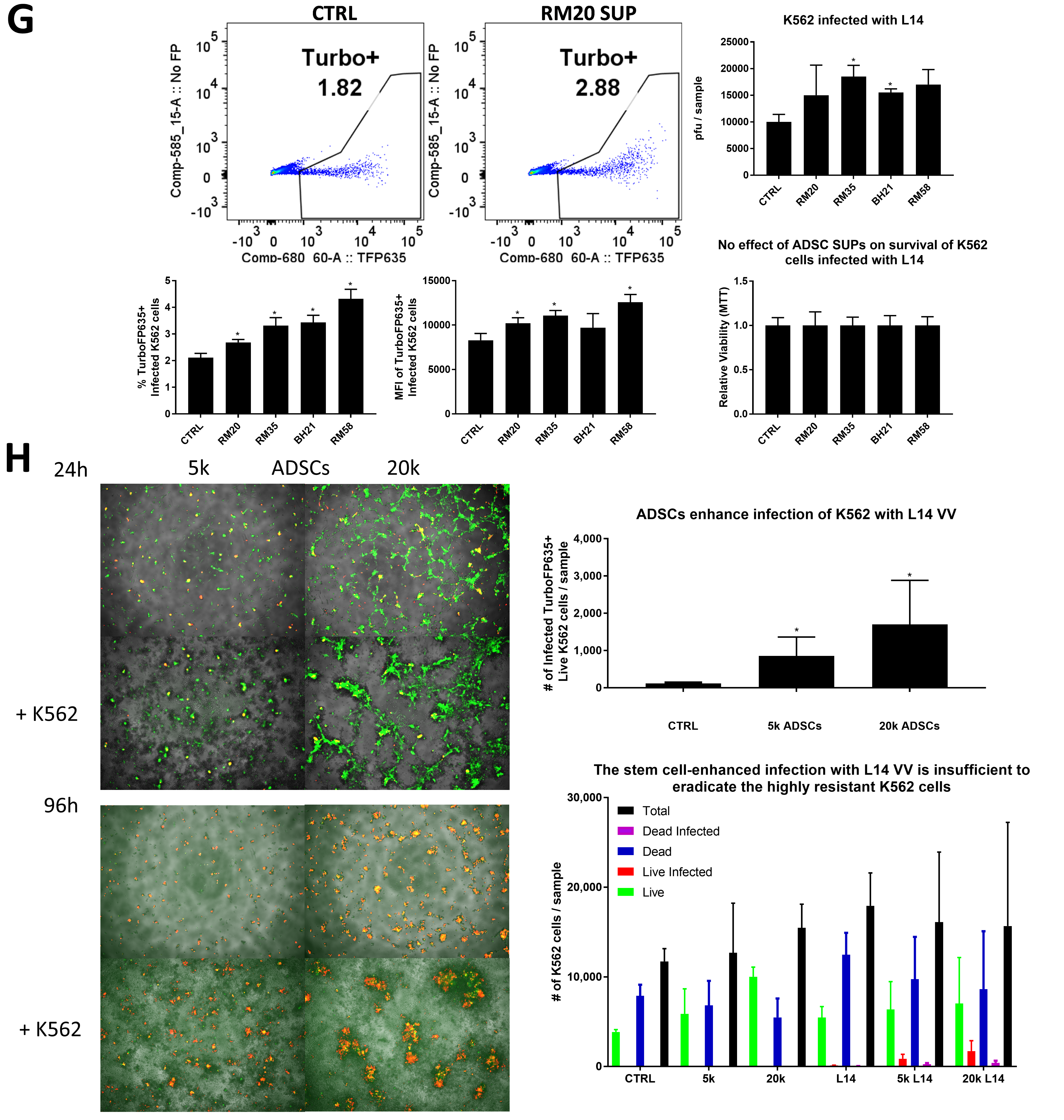

Supplement: Supplementary file 2 — Additional file 2: Figure S2. ADSCs promote the oncolysis of resistant tumor cell lines through a combination of virus amplification, tumor cell recruitment and secretion of factors sensitizing the resistant tumor cells to virus infection. (A) Human ADSC promote the oncolysis of resistant B16 melanoma cells through augmented amplification of the TurboFP635-engineered L14 vaccinia virus. The figure shows fluorescence image analysis of 1 × 10^6^ B16 cells cocultured with 2 × 10^5^ eGFP-labelled RM20 adipose-derived stem cells (4× magnification) in a 12-well plate. B16 and stem cells were infected together with 1 × 10^5^ pfu virus (MOI = 0.1 to B16) and incubated for up to 72 h (data party shown in Fig. 2a). (B) Human RM35 ADSC can also promote the oncolysis of the resistant murine B16 melanoma cells in vitro. Fluorescence imaging analysis of 1 × 10^6^ B16 cells cocultured with 200,000 ADSC and infected with 100,000 pfu L14 VV for up to 4 days. (C) IFNγ pretreatment protects stem cells only in the presence of relatively resistant B16 but not the highly permissive ADSC and A549 cells. 200,000 RM20-eGFP cells (0.2 M) were pretreated with 20 ng/ml IFNγ for 24 h, cocultured with 200,000 (0.2 M) RM20 ADSC, A549 or B16 cells, and infected with the L14 virus as described in (Fig. 2a). Note that IFNγ pretreatment of the stem cells compromised the oncolysis of the B16 monolayer. (D) Insufficient number of stem cells (2% or lower) results in incomplete oncolysis of the B16 monolayer. B16 cells and RM20-eGFP cells were cocultured and infected with L14 as described in (Fig. 2A). To evaluate the role of stem cell number/dose, we compared the oncolysis of the B16 monolayer in the presence of 200,000 (0.2 M) and 20,000 (0.02 M) stem cells. (E) Fluorescence imaging analysis of B16 (10,000) and K562 (100,000) cells infected with L14 virus at MOI of 0.1 for 96 h in 96-well flat-bottom plates in the presence of ADSC supernatants from different stem cell donors as indicated. (F) Plaque assay anal [file 12967_2019_1829_MOESM2_ESM.zip › SF2GH.TIF]

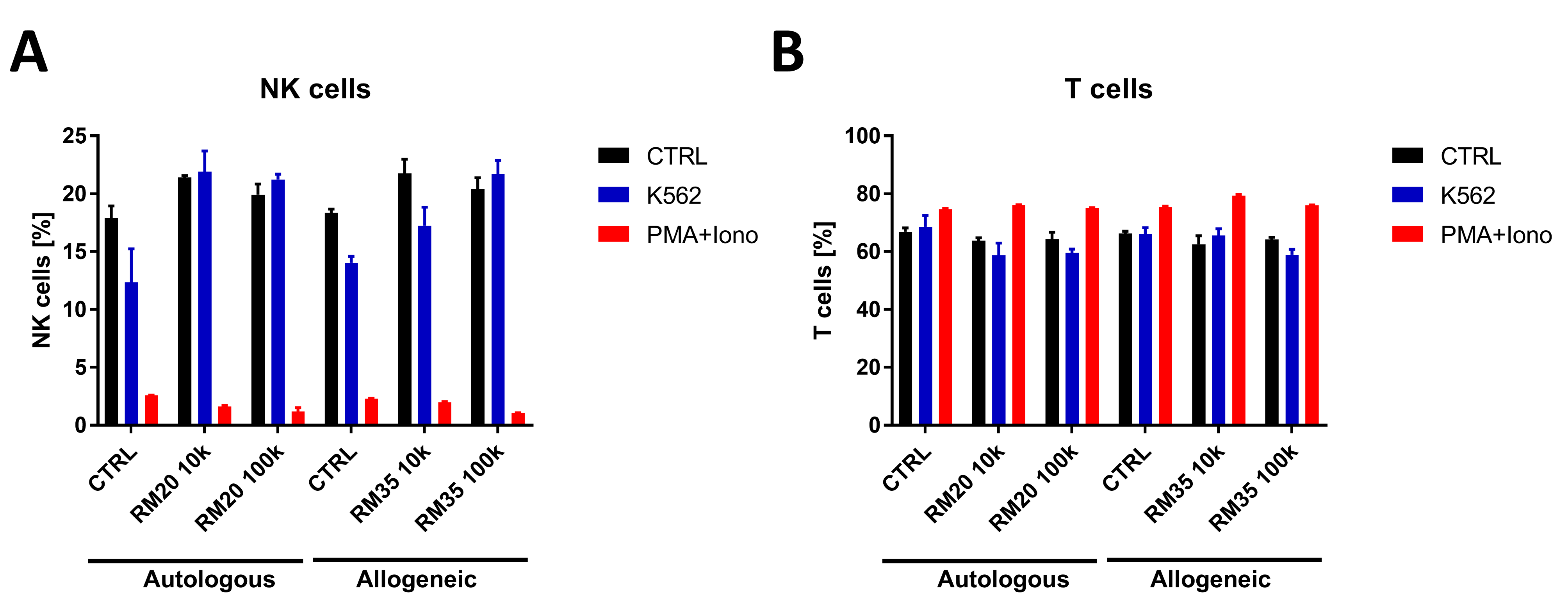

Supplement: Supplementary file 3 — Additional file 3: Figure S3. ADSC are suppressive against NK cells and can overcome allogeneic immune barriers. (A) ADSC-mediated immunosuppression does not affect the frequency of NK and T cells. Note that the PMA-Ionomycin treatment causes downregulation of the NKp46 marker used to identify and gate on NK cells, resulting in “disappearance” of the most activated NK cells. [file 12967_2019_1829_MOESM3_ESM.tif]

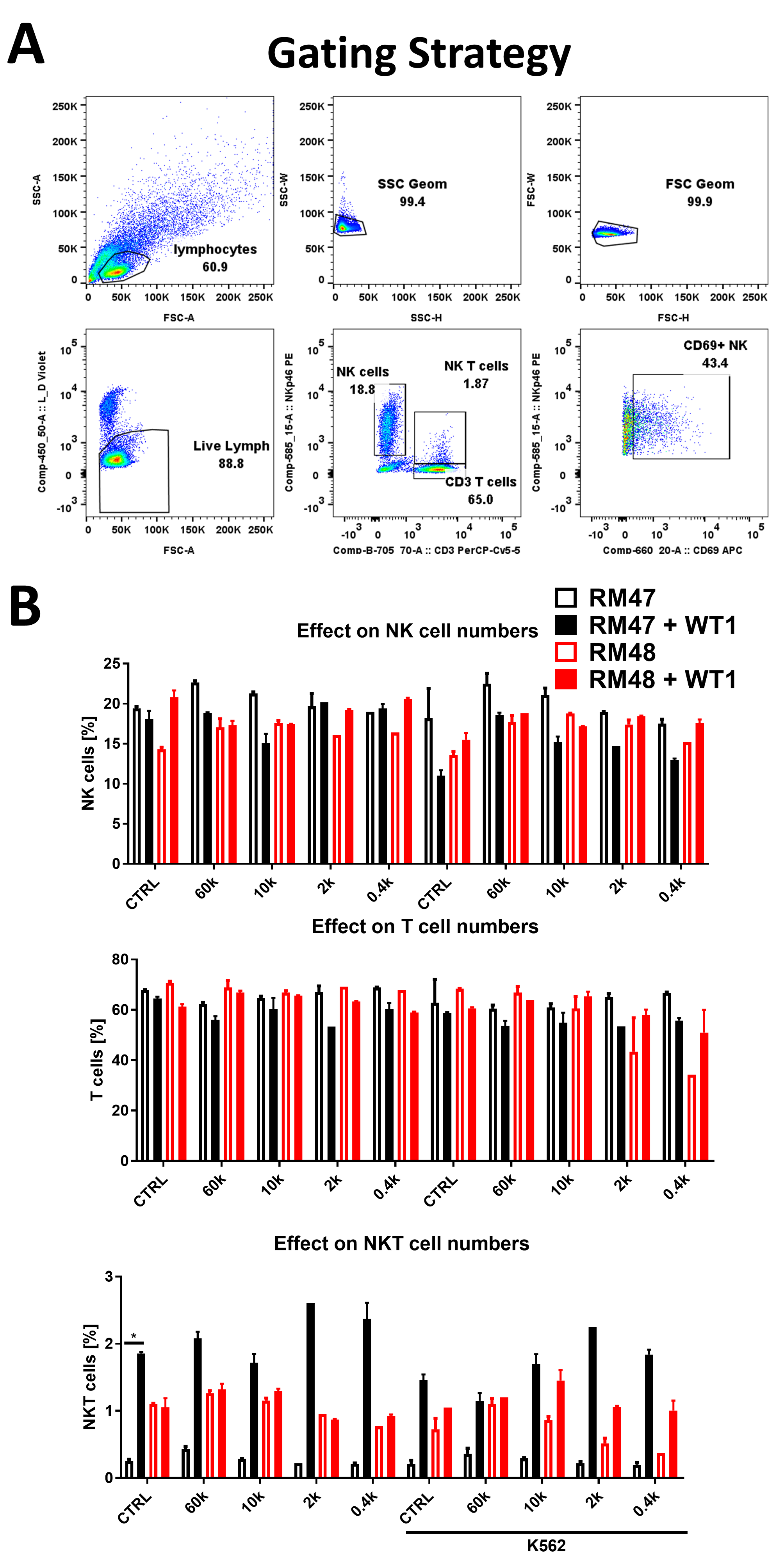

Supplement: Supplementary file 4 — Additional file 4: Figure S4. The potential of allogeneic stem cells to overcome immune barriers correlates with their ability to suppress virus-induced T, NK and NKT cell responses. (A) Gating strategy used to evaluate the effect of ADSC and vaccinia virus on activation of T, NK, and NKT-like cells as measured by upregulation of surface CD69 expression. (B) Summary of the modulation of the three immune cell populations as percentage of gated live lymphocytes in patients RM047 and RM048 (See Fig. 4b). [file 12967_2019_1829_MOESM4_ESM.tif]

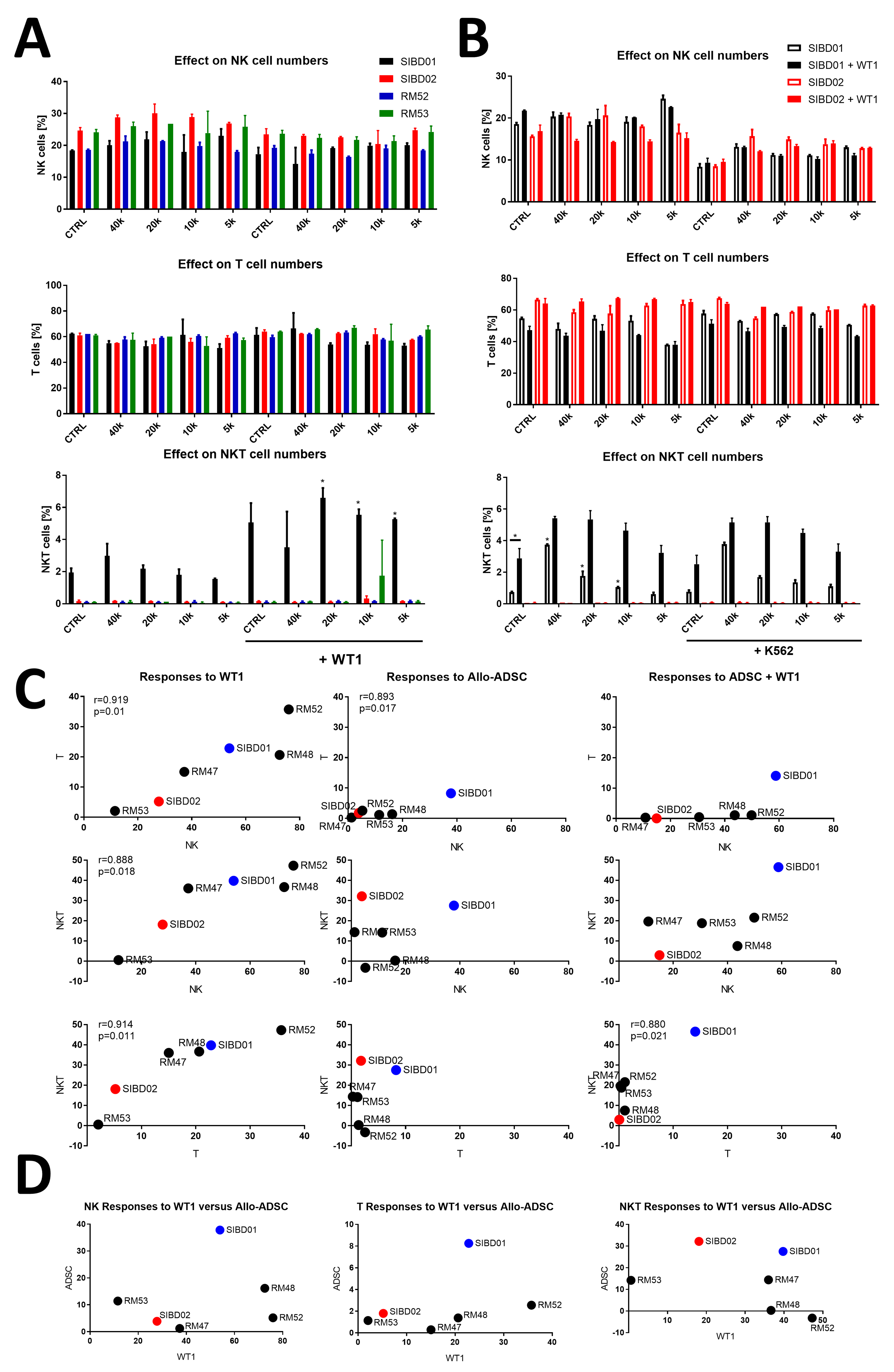

Supplement: Supplementary file 5 — Additional file 5: Figure S5. The potential of allogeneic stem cells to function as a Trojan horse is restricted by patient-specific differences suggesting that proper matching would be required. (A) Patients demonstrate highly variable responses to the allogeneic stem cells and the virus alone or in combination. Flow cytometry analysis of gated live NK, T, and NKT cells from the 48 h cocultures of 250 k PBMCs from 4 different blood donors with 5–40 k allogeneic RM20 ADSC in the presence or absence of 5 k pfu of WT1 VV. Data show the percentage of each gated cell type in PBMC. (B) Flow cytometry analysis of cocultures of PBMC from the SIBD01 and SIBD02 blood donors as in (A) followed by a 4 h stimulation with K562 cells to evaluate the extent of NK cell suppression. (C) Correlative analysis of NK, T, and NKT cell responses (% CD69+ normalized to untreated control) against the WT1 virus, the allogeneic ADSCs or the combo as in F5A, partly shown in F5D. (D) Lack of correlation between NK, T and NKT responsiveness to the virus versus the allogeneic ADSC as in Fig. 5d. Statistically significant differences (Student T-test, p < 0.05) based on duplicates versus corresponding PBMC alone controls (CTRL) or as indicated are marked with asterisks. [file 12967_2019_1829_MOESM5_ESM.tif]

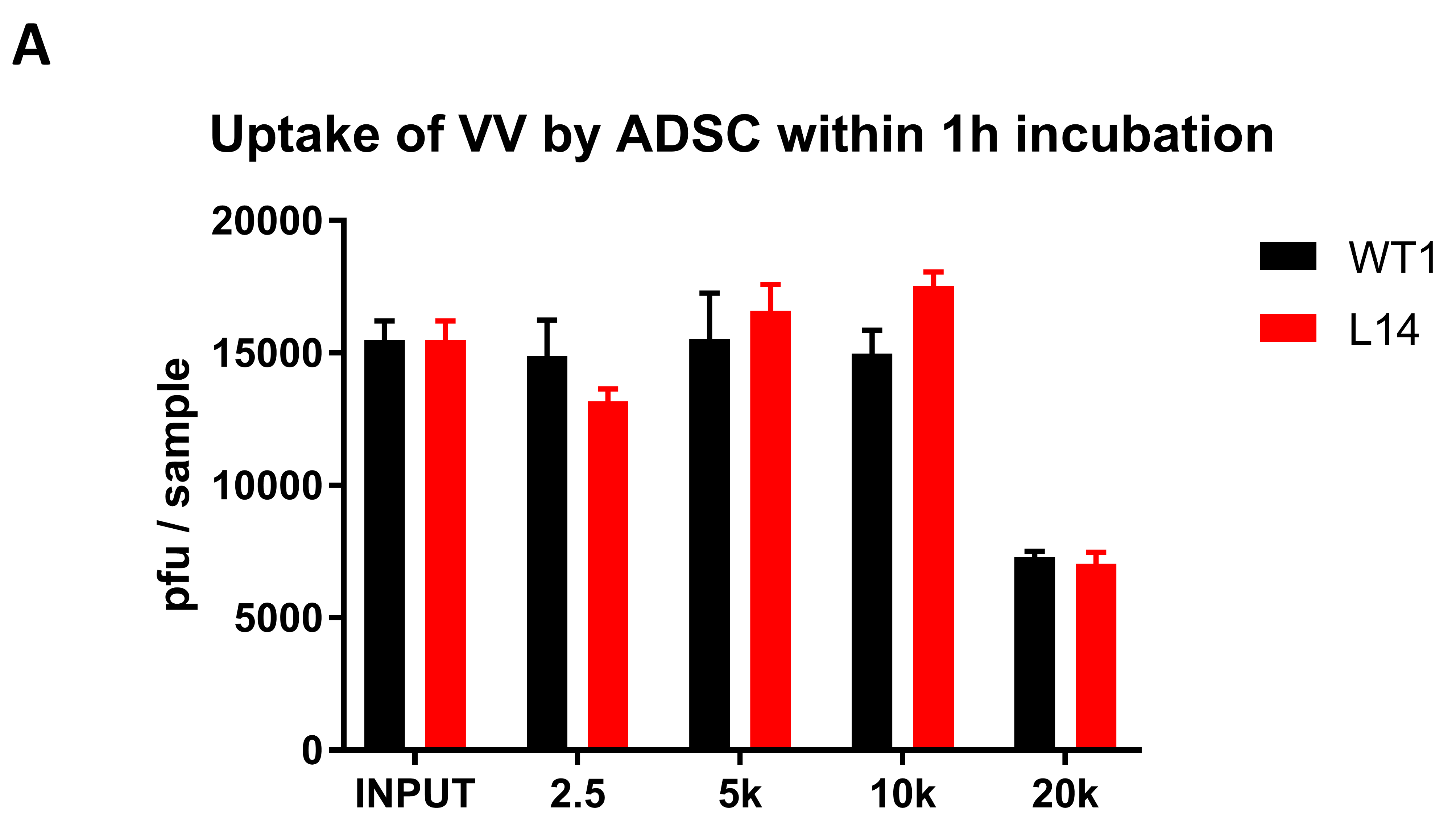

Supplement: Supplementary file 6 — Additional file 6: Figure S6. Patient-specific immunological barriers to the Trojan horse can limit the therapeutic potential of both genetically attenuated and wild type vaccinia virus strains. (A) Plaque analysis of supernatants after 1 h pre-incubation with ADSC in 37 °C incubator with constant shaking showing that at MOI of 1 (Ratio of VV to ADSC = 1) approximately half of the INPUT vaccinia virus gets attached or integrated in the pelleted cells and is absent from the supernatant. At higher MOI (fewer stem cells) most of the virus appears to remain free and requires longer time to integrate. [file 12967_2019_1829_MOESM6_ESM.tif]

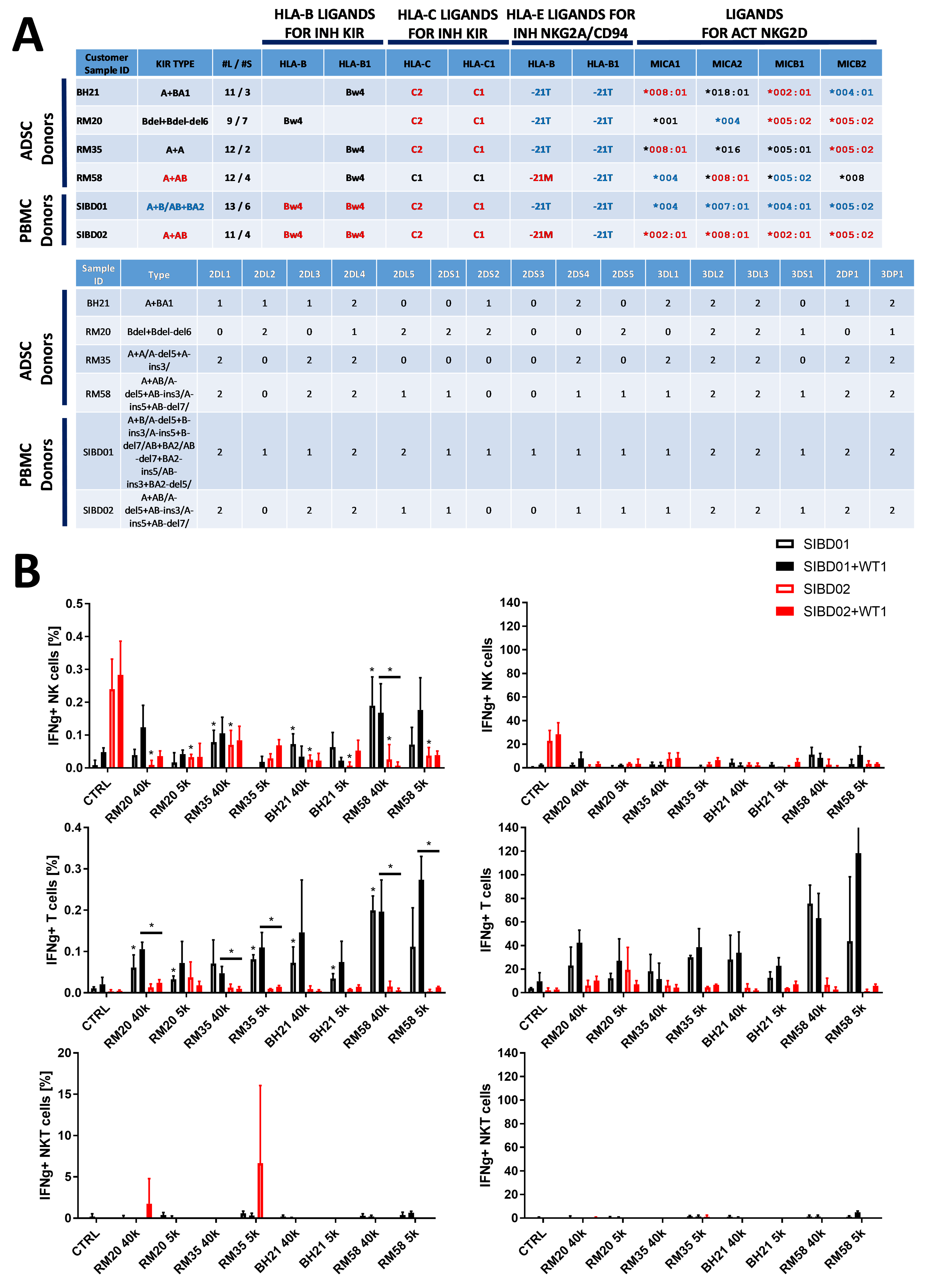

Supplement: Supplementary file 7 — Additional file 7: Figure S7. Patients’ resistance to the Trojan horse is associated with critical HLA mismatches and the rapid induction of anti-stem cell cytotoxic and interferon responses. (A) The top table shows analysis of KIR Haplotypes as well as the presence of known KIR ligands including the Bw4 epitope (HLA-B) and the weak/strong C1/C2 epitopes (HLA-C). This table also includes analysis of the oligomorphic MICA/B molecules that serve as ligands for NKG2D activating receptors on NK cells. The bottom table shows the distribution and copy number of long(L)-inhibitory and short(S)-activating KIR receptors, with the total number of inhibitory and activating receptors present also summarized in the top table. Note the absence of clear correlation between permissiveness/resistance and KIR haplotype/KIR ligands, − 21 M/T dimorphism, and MICA/B oligomorphism. The RM58 stem cells manifest a potentially important KIR ligand C1/C2 mismatch with both the resistant SIBD01 and permissive SIBD02 blood donors, suggesting that such a mismatch alone is insufficient to confer resistance, which might also require additional and stronger HLA mismatching. (B–F) Flow cytometry analysis of gated live NK, NKT and T cells from the PBMC/ADSC/WT1 co-cultures, as in main Fig. 7, showing that all the 4 allogeneic stem cell lines tested induce much stronger CD107α and IFNγ responses in the NK and T cells from the resistant but not permissive blood donor even in the absence of the virus. The figure shows the average frequency and total numbers of IFNγ (B) or CD107α (C) single positive as well as the much lower-frequency IFNγ plus CD107α-double positive lymphocytes of each cell type. (E) Complete correlative analysis of gated live NK, NKT and T cells from the PBMC/ADSC/WT1 co-cultures as above (partly included in main Fig. 7c) showing the average percentages of CD107α or IFNγ single positive lymphocytes of each cell type based on triplicate wells and normalized to respective background (un [file 12967_2019_1829_MOESM7_ESM.zip › SF7AB.TIF]

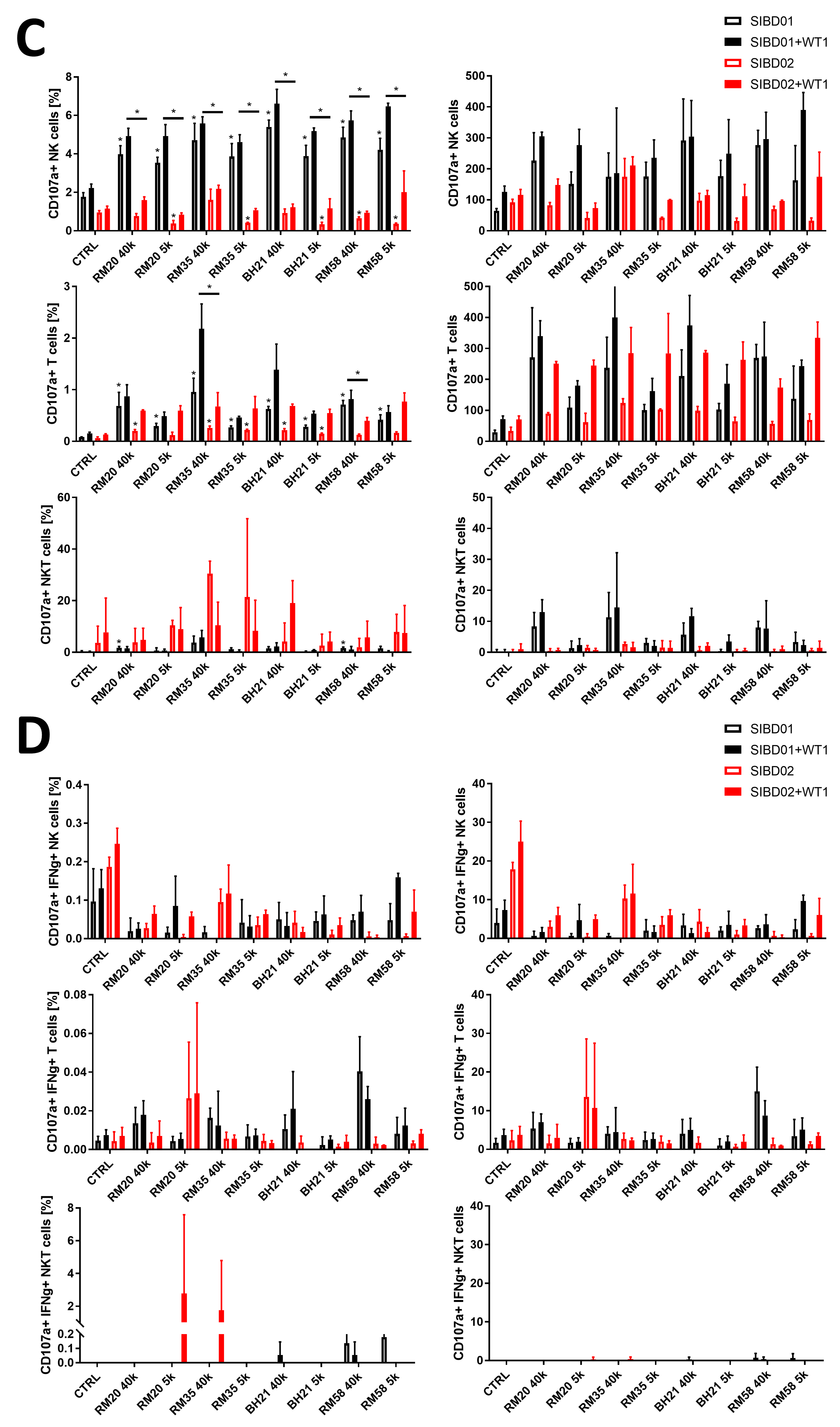

Supplement: Supplementary file 7 — Additional file 7: Figure S7. Patients’ resistance to the Trojan horse is associated with critical HLA mismatches and the rapid induction of anti-stem cell cytotoxic and interferon responses. (A) The top table shows analysis of KIR Haplotypes as well as the presence of known KIR ligands including the Bw4 epitope (HLA-B) and the weak/strong C1/C2 epitopes (HLA-C). This table also includes analysis of the oligomorphic MICA/B molecules that serve as ligands for NKG2D activating receptors on NK cells. The bottom table shows the distribution and copy number of long(L)-inhibitory and short(S)-activating KIR receptors, with the total number of inhibitory and activating receptors present also summarized in the top table. Note the absence of clear correlation between permissiveness/resistance and KIR haplotype/KIR ligands, − 21 M/T dimorphism, and MICA/B oligomorphism. The RM58 stem cells manifest a potentially important KIR ligand C1/C2 mismatch with both the resistant SIBD01 and permissive SIBD02 blood donors, suggesting that such a mismatch alone is insufficient to confer resistance, which might also require additional and stronger HLA mismatching. (B–F) Flow cytometry analysis of gated live NK, NKT and T cells from the PBMC/ADSC/WT1 co-cultures, as in main Fig. 7, showing that all the 4 allogeneic stem cell lines tested induce much stronger CD107α and IFNγ responses in the NK and T cells from the resistant but not permissive blood donor even in the absence of the virus. The figure shows the average frequency and total numbers of IFNγ (B) or CD107α (C) single positive as well as the much lower-frequency IFNγ plus CD107α-double positive lymphocytes of each cell type. (E) Complete correlative analysis of gated live NK, NKT and T cells from the PBMC/ADSC/WT1 co-cultures as above (partly included in main Fig. 7c) showing the average percentages of CD107α or IFNγ single positive lymphocytes of each cell type based on triplicate wells and normalized to respective background (un [file 12967_2019_1829_MOESM7_ESM.zip › SF7CD.TIF]

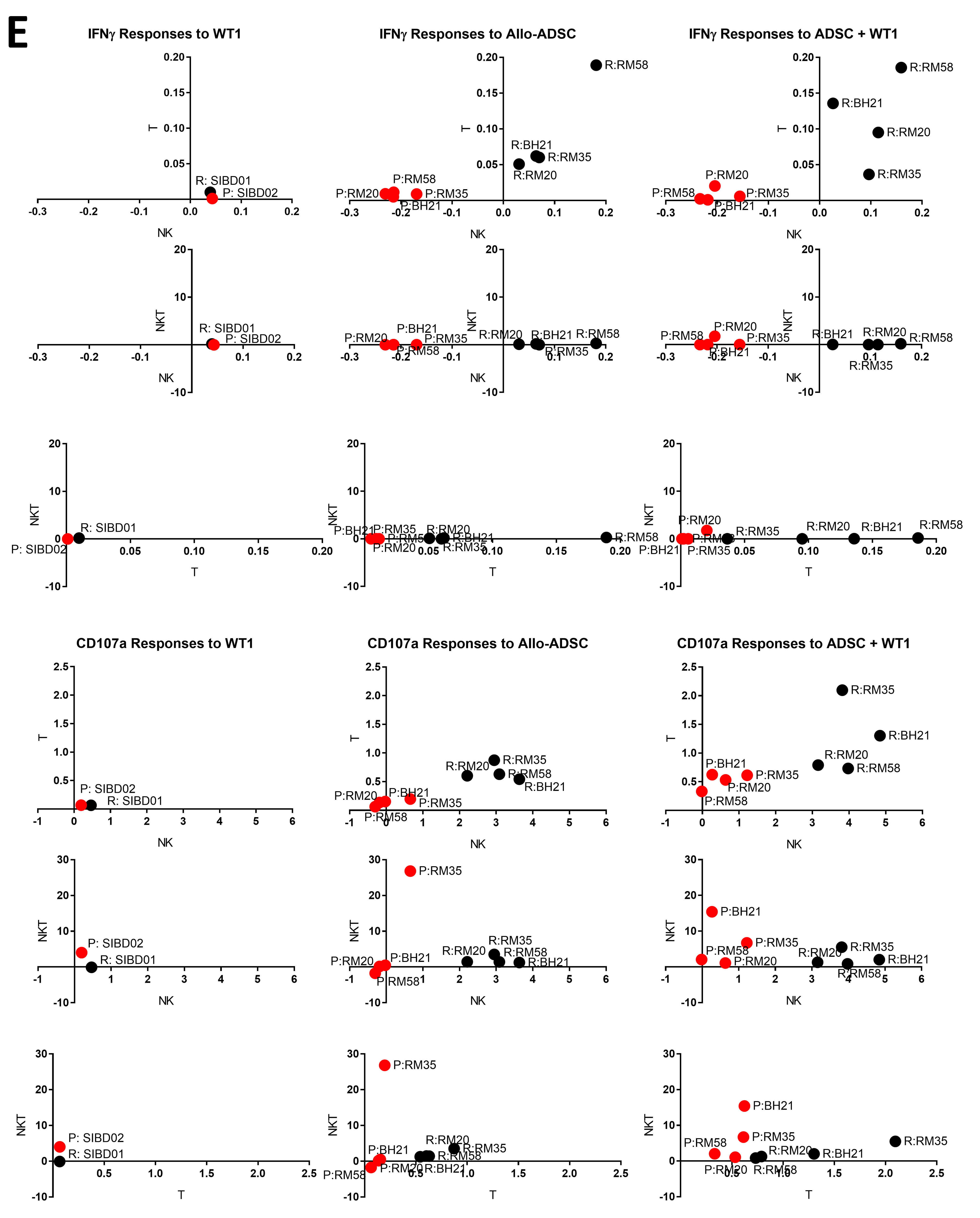

Supplement: Supplementary file 7 — Additional file 7: Figure S7. Patients’ resistance to the Trojan horse is associated with critical HLA mismatches and the rapid induction of anti-stem cell cytotoxic and interferon responses. (A) The top table shows analysis of KIR Haplotypes as well as the presence of known KIR ligands including the Bw4 epitope (HLA-B) and the weak/strong C1/C2 epitopes (HLA-C). This table also includes analysis of the oligomorphic MICA/B molecules that serve as ligands for NKG2D activating receptors on NK cells. The bottom table shows the distribution and copy number of long(L)-inhibitory and short(S)-activating KIR receptors, with the total number of inhibitory and activating receptors present also summarized in the top table. Note the absence of clear correlation between permissiveness/resistance and KIR haplotype/KIR ligands, − 21 M/T dimorphism, and MICA/B oligomorphism. The RM58 stem cells manifest a potentially important KIR ligand C1/C2 mismatch with both the resistant SIBD01 and permissive SIBD02 blood donors, suggesting that such a mismatch alone is insufficient to confer resistance, which might also require additional and stronger HLA mismatching. (B–F) Flow cytometry analysis of gated live NK, NKT and T cells from the PBMC/ADSC/WT1 co-cultures, as in main Fig. 7, showing that all the 4 allogeneic stem cell lines tested induce much stronger CD107α and IFNγ responses in the NK and T cells from the resistant but not permissive blood donor even in the absence of the virus. The figure shows the average frequency and total numbers of IFNγ (B) or CD107α (C) single positive as well as the much lower-frequency IFNγ plus CD107α-double positive lymphocytes of each cell type. (E) Complete correlative analysis of gated live NK, NKT and T cells from the PBMC/ADSC/WT1 co-cultures as above (partly included in main Fig. 7c) showing the average percentages of CD107α or IFNγ single positive lymphocytes of each cell type based on triplicate wells and normalized to respective background (un [file 12967_2019_1829_MOESM7_ESM.zip › SF7E.TIF]

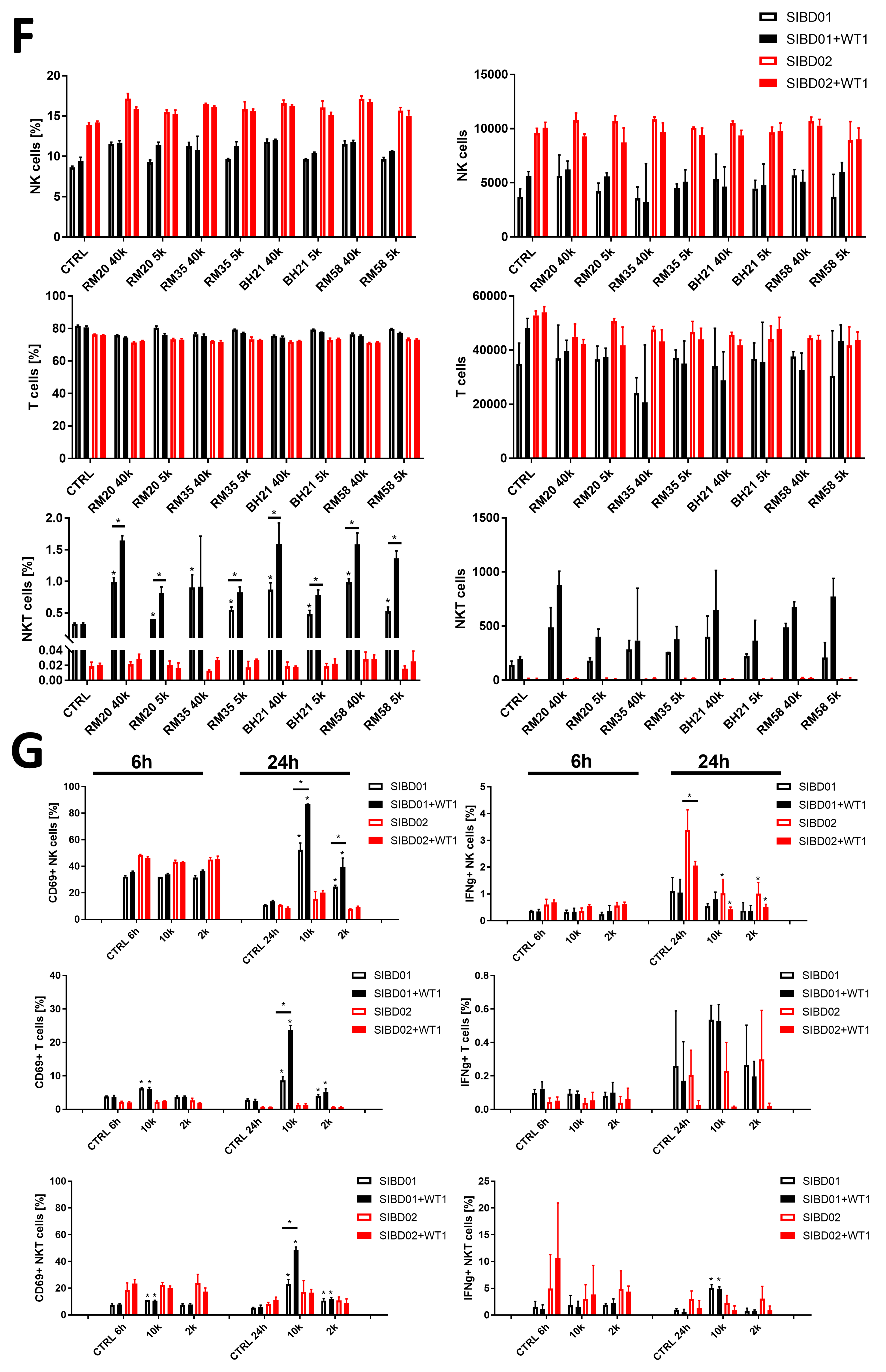

Supplement: Supplementary file 7 — Additional file 7: Figure S7. Patients’ resistance to the Trojan horse is associated with critical HLA mismatches and the rapid induction of anti-stem cell cytotoxic and interferon responses. (A) The top table shows analysis of KIR Haplotypes as well as the presence of known KIR ligands including the Bw4 epitope (HLA-B) and the weak/strong C1/C2 epitopes (HLA-C). This table also includes analysis of the oligomorphic MICA/B molecules that serve as ligands for NKG2D activating receptors on NK cells. The bottom table shows the distribution and copy number of long(L)-inhibitory and short(S)-activating KIR receptors, with the total number of inhibitory and activating receptors present also summarized in the top table. Note the absence of clear correlation between permissiveness/resistance and KIR haplotype/KIR ligands, − 21 M/T dimorphism, and MICA/B oligomorphism. The RM58 stem cells manifest a potentially important KIR ligand C1/C2 mismatch with both the resistant SIBD01 and permissive SIBD02 blood donors, suggesting that such a mismatch alone is insufficient to confer resistance, which might also require additional and stronger HLA mismatching. (B–F) Flow cytometry analysis of gated live NK, NKT and T cells from the PBMC/ADSC/WT1 co-cultures, as in main Fig. 7, showing that all the 4 allogeneic stem cell lines tested induce much stronger CD107α and IFNγ responses in the NK and T cells from the resistant but not permissive blood donor even in the absence of the virus. The figure shows the average frequency and total numbers of IFNγ (B) or CD107α (C) single positive as well as the much lower-frequency IFNγ plus CD107α-double positive lymphocytes of each cell type. (E) Complete correlative analysis of gated live NK, NKT and T cells from the PBMC/ADSC/WT1 co-cultures as above (partly included in main Fig. 7c) showing the average percentages of CD107α or IFNγ single positive lymphocytes of each cell type based on triplicate wells and normalized to respective background (un [file 12967_2019_1829_MOESM7_ESM.zip › SF7FG.TIF]

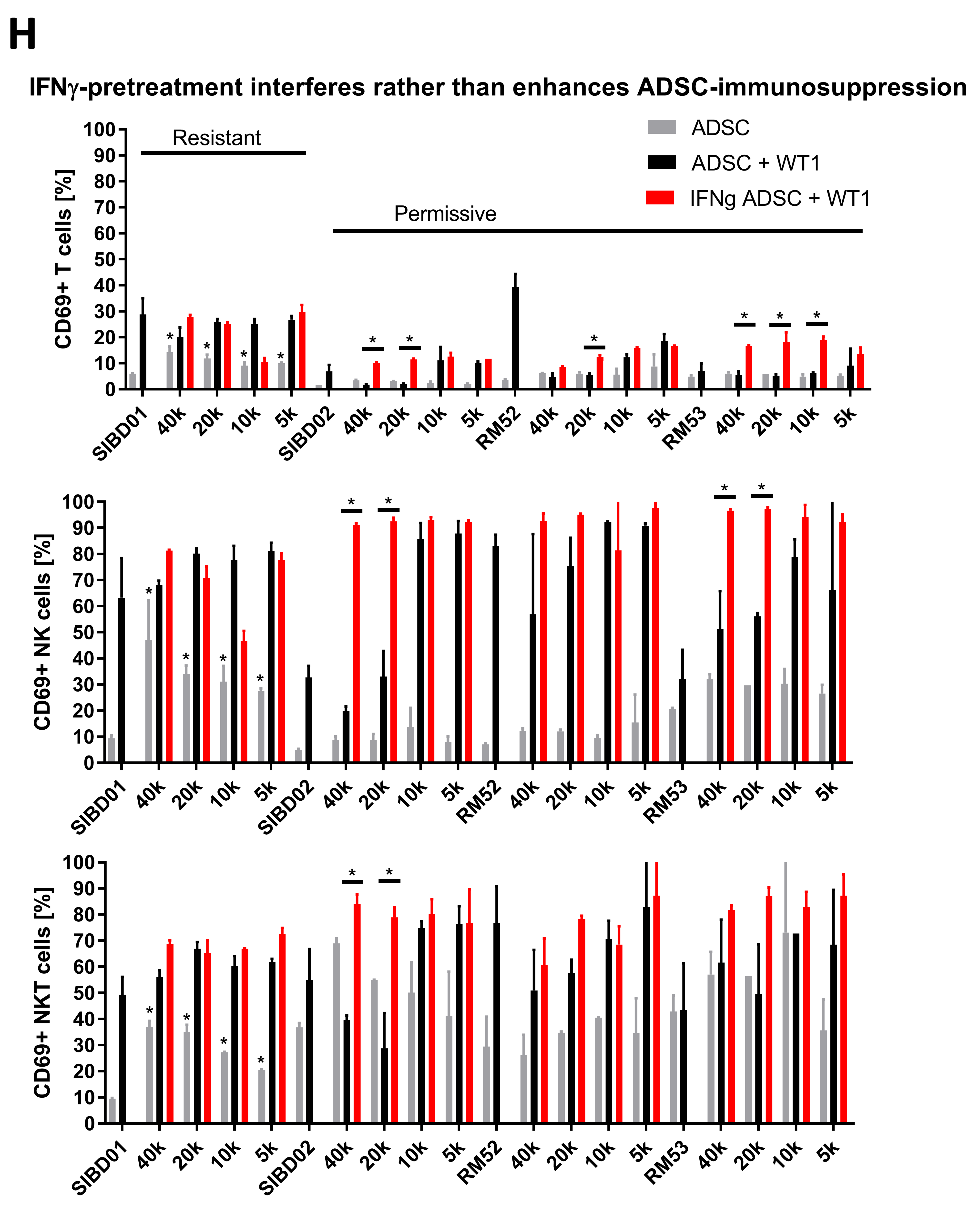

Supplement: Supplementary file 7 — Additional file 7: Figure S7. Patients’ resistance to the Trojan horse is associated with critical HLA mismatches and the rapid induction of anti-stem cell cytotoxic and interferon responses. (A) The top table shows analysis of KIR Haplotypes as well as the presence of known KIR ligands including the Bw4 epitope (HLA-B) and the weak/strong C1/C2 epitopes (HLA-C). This table also includes analysis of the oligomorphic MICA/B molecules that serve as ligands for NKG2D activating receptors on NK cells. The bottom table shows the distribution and copy number of long(L)-inhibitory and short(S)-activating KIR receptors, with the total number of inhibitory and activating receptors present also summarized in the top table. Note the absence of clear correlation between permissiveness/resistance and KIR haplotype/KIR ligands, − 21 M/T dimorphism, and MICA/B oligomorphism. The RM58 stem cells manifest a potentially important KIR ligand C1/C2 mismatch with both the resistant SIBD01 and permissive SIBD02 blood donors, suggesting that such a mismatch alone is insufficient to confer resistance, which might also require additional and stronger HLA mismatching. (B–F) Flow cytometry analysis of gated live NK, NKT and T cells from the PBMC/ADSC/WT1 co-cultures, as in main Fig. 7, showing that all the 4 allogeneic stem cell lines tested induce much stronger CD107α and IFNγ responses in the NK and T cells from the resistant but not permissive blood donor even in the absence of the virus. The figure shows the average frequency and total numbers of IFNγ (B) or CD107α (C) single positive as well as the much lower-frequency IFNγ plus CD107α-double positive lymphocytes of each cell type. (E) Complete correlative analysis of gated live NK, NKT and T cells from the PBMC/ADSC/WT1 co-cultures as above (partly included in main Fig. 7c) showing the average percentages of CD107α or IFNγ single positive lymphocytes of each cell type based on triplicate wells and normalized to respective background (un [file 12967_2019_1829_MOESM7_ESM.zip › SF7H.TIF]

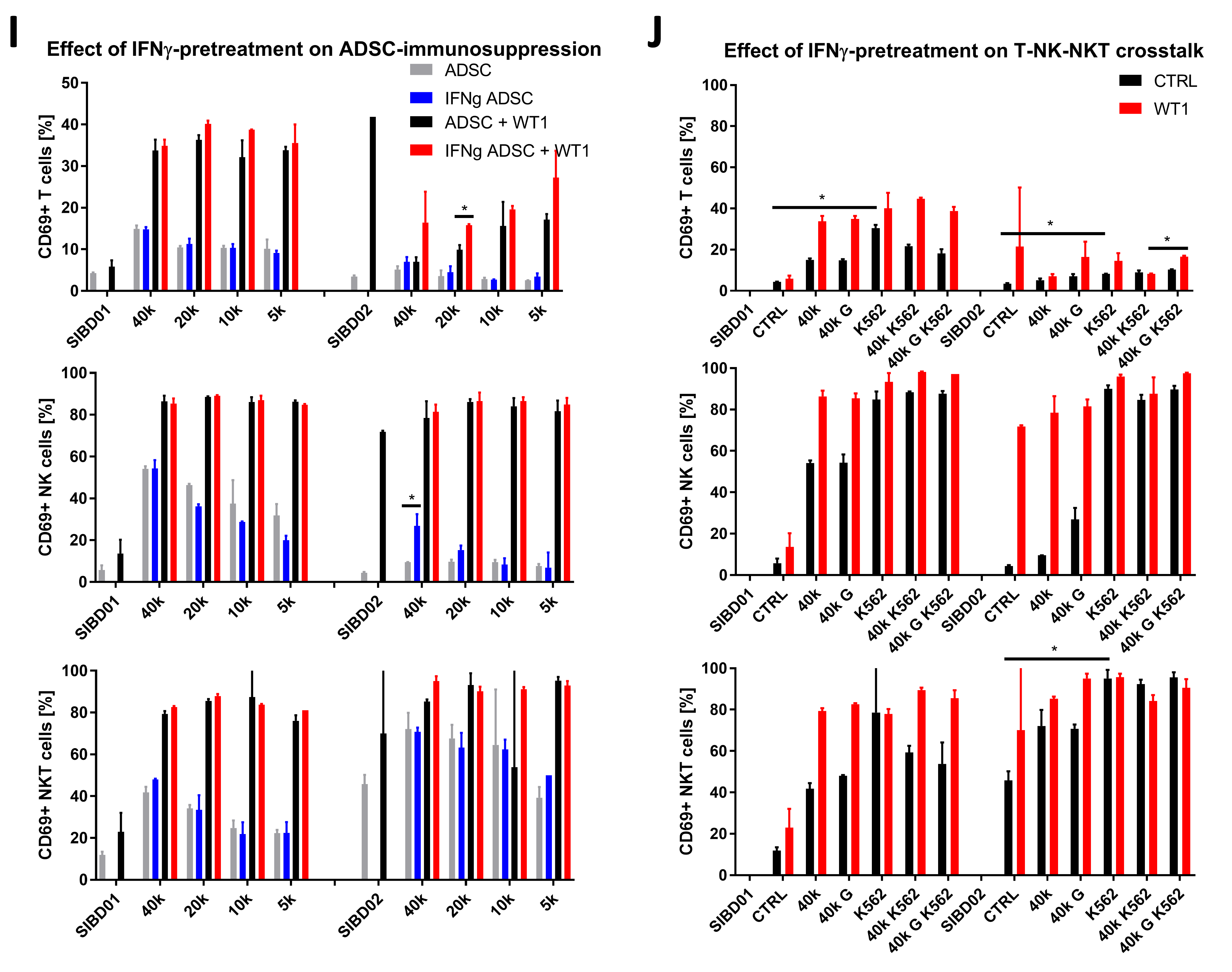

Supplement: Supplementary file 7 — Additional file 7: Figure S7. Patients’ resistance to the Trojan horse is associated with critical HLA mismatches and the rapid induction of anti-stem cell cytotoxic and interferon responses. (A) The top table shows analysis of KIR Haplotypes as well as the presence of known KIR ligands including the Bw4 epitope (HLA-B) and the weak/strong C1/C2 epitopes (HLA-C). This table also includes analysis of the oligomorphic MICA/B molecules that serve as ligands for NKG2D activating receptors on NK cells. The bottom table shows the distribution and copy number of long(L)-inhibitory and short(S)-activating KIR receptors, with the total number of inhibitory and activating receptors present also summarized in the top table. Note the absence of clear correlation between permissiveness/resistance and KIR haplotype/KIR ligands, − 21 M/T dimorphism, and MICA/B oligomorphism. The RM58 stem cells manifest a potentially important KIR ligand C1/C2 mismatch with both the resistant SIBD01 and permissive SIBD02 blood donors, suggesting that such a mismatch alone is insufficient to confer resistance, which might also require additional and stronger HLA mismatching. (B–F) Flow cytometry analysis of gated live NK, NKT and T cells from the PBMC/ADSC/WT1 co-cultures, as in main Fig. 7, showing that all the 4 allogeneic stem cell lines tested induce much stronger CD107α and IFNγ responses in the NK and T cells from the resistant but not permissive blood donor even in the absence of the virus. The figure shows the average frequency and total numbers of IFNγ (B) or CD107α (C) single positive as well as the much lower-frequency IFNγ plus CD107α-double positive lymphocytes of each cell type. (E) Complete correlative analysis of gated live NK, NKT and T cells from the PBMC/ADSC/WT1 co-cultures as above (partly included in main Fig. 7c) showing the average percentages of CD107α or IFNγ single positive lymphocytes of each cell type based on triplicate wells and normalized to respective background (un [file 12967_2019_1829_MOESM7_ESM.zip › SF7IJ.TIF]
